# Developmental validation of GlobalFiler™ PCR amplification kit: a 6-dye multiplex assay designed for amplification of casework samples

**DOI:** 10.1007/s00414-018-1817-5

**Published:** 2018-03-09

**Authors:** Matthew J. Ludeman, Chang Zhong, Julio J. Mulero, Robert E. Lagacé, Lori K. Hennessy, Marc L. Short, Dennis Y. Wang

**Affiliations:** 1 Thermo Fisher Scientific Inc., 180 Oyster Point Blvd, South San Francisco, CA 94080 USA; 2 Spring Bioscience, 4300 Hacienda Dr., Pleasanton, CA 94588 USA

**Keywords:** GlobalFiler™ kit, Forensic validation studies, STR, Multiplex PCR assay, Casework samples, CODIS, ESS

## Abstract

The GlobalFiler™ PCR Amplification Kit is a single multiplex assay that amplifies a set of 24 markers, which encompass the European Standard Set and CODIS (Combined DNA Index System) recommended composite set of loci. In addition to more loci and a 6-dye chemistry format, the Master Mix has been formulated to allow higher sample loading volume for trace DNA samples. The GlobalFiler™ Kit has been optimized to deliver high performance on casework samples, while also delivering fast thermal cycling, with an amplification time of approximately 80 min. Here, we report the results of the developmental validation study which followed the SWGDAM (Scientific Working Group on DNA Analysis Methods) guidelines and includes data for PCR-based studies, sensitivity, species specificity, stability, precision, reproducibility and repeatability, concordance, stutter, DNA mixtures, and performance on mock casework samples. The results validate the multiplex design as well as demonstrate the kit’s robustness, reliability, and suitability as an assay for human identification with casework DNA samples.

**Electronic supplementary material:**

The online version of this article (10.1007/s00414-018-1817-5) contains supplementary material, which is available to authorized users.

## Introduction

Genotyping by targeting short tandem repeats (STRs) present in the human genome has proven to be an extremely discriminating method for human identification in forensic and paternity applications for decades now [1]. With an ever-growing number of STR profiles being generated globally, there has been great interest in ensuring that methods are available to maintain high levels of discriminatory power to reduce the likelihood of adventitious matches, and to enable agencies to share information internationally as effectively as possible [2–5]. This is reflected in the expansion of the CODIS (Combined DNA Index System) loci set required for the U.S., from its longstanding original 13 markers to 20 markers, implemented in January 2017 [6, 7].

The GlobalFiler™ Kit is a multiplex assay that combines the 13 original CODIS loci with 7 non-overlapping loci from the expanded European Standard Set (ESS), as well as the highly discriminating SE33 locus, two Y-based loci and the sex determining maker, Amelogenin [5, 8–14]. The full complement of loci in the GlobalFiler™ Kit are: D13S317, D7S820, D5S818, CSF1PO, D1S1656, D12S391, D2S441, D10S1248, D18S51, FGA, D21S11, D8S1179, vWA, D16S539, TH01, D3S1358, AMEL, D2S1338, D19S433, DYS391, TPOX, D22S1045, SE33 and a Y-specific insertion/deletion locus (Y_indel_).

The GlobalFiler™ Kit has been fully optimized for use on casework samples, with a Master Mix configuration designed to maximize the volume available for sample input. Additionally, the Master Mix has been developed to deliver enhanced sensitivity and inhibitor tolerances, while also being optimized for fast thermal cycling that enables completion of amplification in approximately 80 min [13]. In this article, we report findings that demonstrate the robustness of the GlobalFiler™ chemistry and its suitability for use in the analysis of forensic casework samples. Experiments in the developmental validation studies described here were performed in accordance with guidelines published by the Scientific Working Group on DNA Analysis Methods (SWGDAM) [15].

## Materials and methods

### Human DNA samples

AmpFLSTR™ Control DNA 007 was sourced from Thermo Fisher Scientific (South San Francisco, CA). Control DNAs 9947A, 9948 and Raji were purchased from Marligen Biosciences (Ijamsville, MD), Coriell Cell Repositories (Camden, NJ), and Biochain Institute (Hayward, CA), respectively.

Whole human blood samples from 1194 donors were obtained from the Interstate Blood Bank (Memphis, TN) and Boca Biolistics (Coconut Creek, FL) (see Compliance with Ethical Standards section). The donor samples were collected in the United States from unrelated individuals of self-reported ethnicities. In population studies, the DNA was extracted from blood samples using an ABI™ PRISM 6100 Nucleic Acid PrepStation (Thermo Fisher Scientific).

Artificially degraded DNA samples were prepared in-house by incubating sonicated Raji DNA for 20 min with DNase I enzyme (Ambion Inc., Austin TX) at varying concentrations (0–6 U).

In the bone sample study, DNA was isolated using the PrepFiler Express BTA™ Forensic DNA Extraction Kit (Thermo Fisher Scientific) and the AutoMate Express™ Nucleic Acid Extraction System (Thermo Fisher Scientific). The samples were taken from a human bone specimen (The Bone Room, Berkeley CA) of undetermined age, but known to possess degraded DNA based on earlier analysis with the small-amplicon-based AmpFLSTR™ MiniFiler™ PCR Amplification Kit (Thermo Fisher Scientific). The DNA was quantified using the Quantifiler™ Duo DNA Quantification Kit on an Applied Biosystems™ 7500 Real-Time PCR System (Thermo Fisher Scientific).

A small set of mock casework (non-bone) samples were also generated and extracted in-house. Unless otherwise noted, these samples were extracted using the PrepFiler™ Express Forensic DNA Extraction Kit (Thermo Fisher Scientific) and the AutoMate Express™ Nucleic Acid Extraction System. Swabs used were standard cotton-tipped from Puritan Medical Products Company (Guilford, ME) unless otherwise identified as flocked swabs, which were 4N6FLOQSwabs from Copan Flock Technologies S.r.l. (Brescia, Italy). Soda cans, used by a single, known donor (informed consent obtained), were swabbed around the opening to collect trace DNA. Discarded and found cigarette butts from smoked cigarettes were collected from anonymous donors; the paper from the butt section was removed, cut in half and extracted with PrepFiler Express BTA™ Forensic DNA Extraction system. For “Blood on Soil” samples, whole blood from a previously genotyped donor stock (purchased material, donor # IB-0006) was spotted on small pieces of prewashed white cotton cloth (from Serological Research Institute (Richmond, CA)) and either extracted immediately (Day 0 control) or placed in soil inside a small sterile plastic bottle then left in an incubator at 37 °C for indicated number of days before DNA extraction—for extraction, a single 5 mm punch (fully covered) from each sample was used. For “Blood on Wood/Metal” samples, 5 μL of blood from a previously genotyped, purchased donor stock was applied to a cotton swab and then dabbed onto either the metal or wood portion of hammer. After the sample had been left to dry overnight, a moist cotton swab was used to collect DNA/blood from dabbed areas and then was extracted. For “Semen on Cloth” samples, semen (2 μL) from purchased stocks (Serological Research Institute (Richmond, CA)) was spotted on 5 mm punches of white cotton and either extracted immediately (Day 0 control) or subject to 6 h of UV exposure in a biosafety cabinet before extraction. Mock sample DNA was quantified using the Quantifiler™ Trio DNA Quantification Kit (Thermo Fisher Scientific).

For human DNAs in general, quantification was carried out using either the Quantifiler™ Duo or Quantifiler™ Trio DNA Quantification Kit on an Applied Biosystems™ 7500 Real-Time PCR System. Concentration of non-human DNA was determined by measuring the absorbance of the sample at 260 nm.

### PCR amplification and thermal cycling conditions

Standard condition amplification reactions were 25 μL in total volume, consisting of 7.5 μL of Master Mix, 2.5 μL of Primer Set and 15 μL of sample input. Samples were amplified in MicroAmp™ Optical 96-well reaction plates (Thermo Fisher Scientific) in Applied Biosystems GeneAmp™ PCR system 9700 with a gold-plated silver or silver block under the “Max” ramping mode or Veriti™ 96-well Thermal Cyclers at the 100% ramping rate (all Thermo Fisher Scientific). Standard thermal cycling parameters were as follows: enzyme activation at 95 °C for 1 min; 29 cycles of denaturation at 94 °C for 10 s and annealing / extension at 59 °C for 90 s; followed by a final extension step at 60 °C for 10 min.

### Sample electrophoresis and data analysis

Capillary electrophoresis (CE) of amplification products was performed on Applied Biosystems 3130*xl*, 3500 or 3500xL Genetic Analyzers using run modules and J6 6-dye variable binning modules as described in GlobalFiler™ Kit User Guide [13]. When not specified, the instrument used in these studies was a 3500xL. Samples were prepared for CE by adding 1 μL of the PCR product (or GlobalFiler™ Allelic Ladder) to 10 μL of formamide / size standard solution (9.6 μL of deionized Hi-Di™ Formamide plus 0.4 μL of GeneScan™ 600 LIZ™ Size Standard v2.0) (Thermo Fisher Scientific). Just prior to electrophoresis, the samples were denatured at 95 °C for 3 min then chilled on wet ice. Samples were then injected at 1.2 kV for 24 s. and electrophoresed at 13 kV for 1550 s. in Performance Optimized Polymer-4 (POP-4 polymer) (Thermo Fisher Scientific). Unless otherwise noted, after data collection, CE results were analyzed using GeneMapper™ *ID-X* Software v1.4 (Thermo Fisher Scientific), with a threshold for allele peak calls set at 175 relative fluorescence units (RFU) and a kit-specific read region of 74–444 nt.

### PCR primer set and master mix components

The GlobalFiler™ Kit contains PCR primer sets for 24 loci. Coverage of 22 of these loci has been accomplished with existing primer sequences utilized in the NGM SElect™ or Identifiler™ Plus Kits [13]. This suite of existing primer sets includes supplemental degenerate SNP-specific primers for vWA, D16S539, Amel, D2S441, D22S1045, and D8S1179 loci [16, 17]. Beyond this, primers for a novel Y_indel_ have been added. Additionally, to capture other rare allelic variants more recently identified, new degenerate SNP-specific primers have been added to the GlobalFiler™ Kit for D3S1358, vWA, D18S51, D19S433, TH01, FGA, D5S819 and SE33 [13]. One primer for TPOX and DYS391 has also been redesigned from Identifiler™ Plus and Yfiler™ Kits, respectively, to increase the size of the amplicon and facilitate optimal marker spacing [14, 17]. The 5 dyes used in the GlobalFiler™ Kit to label amplified sample products are 6-FAM™, VIC™, NED™, TAZ™, and SID™. The sixth dye, LIZ™, is used to label the GeneScan™ 600 LIZ™ Size Standard v2.0 (Thermo Fisher Scientific).

The PCR Master Mix components of the GlobalFiler™ Master Mix include a “hot-start” DNA polymerase, buffer, salts, dNTPs, detergent, carrier protein, sodium azide and stabilizing components. From an optimized formulation, reagents and individual Master Mix components were varied at 10% increments up to +/− 30% (*v*/*v*) from the standard formulation to test for reliability and robustness of the Master Mix formulation. Three genomic DNA samples (Control DNA 9947A, 9948, and 007) were evaluated in triplicate for each component at each concentration. The initial release of the GlobalFiler™ Kit included a Master Mix raw material in the form of an Additive that was required to be added to the GlobalFiler™ Master Mix post-thaw and prior to first use. The separation of the PCR Master Mix and the PCR Additive was due to incompatibility of PCR buffer formulation for long term storage under freezing condition. Subsequently, the incompatibility of the raw material was resolved in 2015 and the GlobalFiler™ Master Mix was updated to a single tube format, providing for greater ease of use while maintaining desired cold-storage capabilities. Some of the experiments done during developmental validation of the kit and presented here predate that change; however, the verification study of this raw material format change is well documented in the current GlobalFiler™ User Guide [13] and functional equivalence of Master Mix formulations is extensively demonstrated there, as well as in results presented in this work (see Stutter section below).

### Species specificity

Non-human DNA samples from chimpanzee, orangutan, and macaque were purchased in purified form from BIOS Laboratories (New Haven, CT). Non-primate whole blood samples were obtained from Pel-Freez Biologicals (Rogers, AK) and their genomic DNA was extracted and purified using the ABI™ PRISM 6100 Nucleic Acid PrepStation. Pooled genomic DNAs from several human-associated microbial species (c. 10^5^ copies each from *Candida albicans, Staphylococcus aureus, Escherichia coli, Neisseria gonorrhoeae, Bacillus subtilis*, and *Lactobacillus rhamnosus*) obtained from American Type Culture Collection (Manassas, VA) were prepared from cultures grown and purified in-house with the Iso-Quick Nucleic Acid Extraction Kit (Orca Research, Inc., Bothell, WA). The specificity of the PCR amplification with the GlobalFiler™ Kit for human DNA was assessed with DNA samples from primates (1 ng each), non-primate animals (10 ng each), and pooled microorganisms (10 ng). Amplified products were then electrophoresed on the 3500xL instrument and analyzed for above-background peaks in or near the read region with GeneMapper™ *ID-X* 1.4 software.

### Inhibition models

Model systems were used to test stability of the assay by examining performance in the presence of known PCR-inhibitory substances that can be commonly encountered in forensic casework samples, or as carry-through from upstream sample preparation/processing [18, 19]. In these experiments, test substances were added directly to STR reactions, after which, amplification, electrophoresis and analysis was carried out under standard conditions. Hematin, humic acid, and phenol-chloroform were added to reactions, after being prepared as described here. Stock solutions of high concentration were prepared by dissolving hematin (Sigma, St. Louis, MO) in 0.1 N NaOH and humic acid (Sigma) in water, after which, both were further diluted in water to make working stocks as appropriate. To avoid potential non-homogeneity of the phenol-chloroform (phenol/chloroform/isoamyl alcohol 25:24:1) (Sigma), which could possibly occur in working stocks or reaction mix batches, an aliquot of the stock solution was taken, maintained in a mixed state with periodic pulse vortexing and delivered undiluted in low volumes directly into each reaction mixture just prior to amplification.

### Sensitivity and performance over a range of DNA inputs

Serial dilutions of Control DNA 007 were analyzed with GlobalFiler™ and Identifiler™ Plus Kits. To test for sensitivity, four replicate reactions were run with the following inputs: 1000, 500, 250, 125, 62.5, 31.2 and 15.6 pg DNA (total per reaction). Non-template controls (or, NTCs) were also run. To test the effects of *high* DNA input specifically on GlobalFiler™ performance, four replicate runs each with Control DNA 007 input of 1 ng, 2 ng and 3 ng were also run. Amplified products were electrophoresed on the 3500xL instrument and analyzed with GeneMapper™ *ID-X* 1.4 software.

### Degraded DNA

Samples of progressively degraded DNA (described above), along with a non-degraded controls were analyzed with GlobalFiler™ and Identifiler™ Plus Kits in triplicate under otherwise standard reaction conditions.

### DNA mixtures

DNA from two control samples, Raji and Control 007 DNA (see Online Resource 1 for Mixture Study genotypes), was used. Standard GlobalFiler™ Kit reaction conditions were used and total combined template DNA input was maintained at 1.0 ng for all mixture reactions, which were run in triplicate at ratios of 1:1, 1:5 and 1:8, with control runs (1:0 and 0:1, non-mixture DNA) for both minor and major contributors. Amplified products were then electrophoresed on the 3500xL instrument and analyzed with GeneMapper™ *ID-X* 1.4 software.

### Bone sample

DNA was isolated from a human bone specimen known to possess degraded DNA (see Human DNA section above). The DNA was extracted and quantified as described above, and input for reactions with all kits was approximately 1.0 ng total (4 μL of isolated sample DNA (approximate eluate concentration of 0.25 ng/μL)) supplemented with appropriate amount of diluent buffer. Allele calls were tallied and deemed to be correct based on previous analysis of DNA from this sample using the MiniFiler™ Kit.

### Mock casework samples

DNA from mock casework samples was extracted and quantified as described above, then run with GlobalFiler™ and Identifiler™ Plus Kits. DNA inputs differed in the case of highly dilute samples (< 0.1 ng/μL), as the two kits have different maximum sample input volumes (15uL and 10uL, for GlobalFiler™ and Identifiler™ Plus Kits, respectively). Electropherogram profiles were analyzed and total alleles recovered for each sample were tabulated. Genotypes were known for all DNA samples in this study, with the exception of the three cigarette butt samples. For these samples, all allele calls at individual loci were cross-checked whenever possible between the two kits above as well as a third prototype multiplex kit that interrogates a sub-set of alleles common to GlobalFiler™ and Identifiler kits with novel primer pairs, to eliminate or minimize potential spurious allele calls. No counted alleles were discordant between the three multiplexes or with known genotypes, as applicable.

### Stutter effects

Sutter effects with the GlobalFiler™ assay were examined as part of a larger study that also encompassed allele sizing accuracy and precision. Results on allele sizing accuracy and precision can be found in the User Guide [13].

Minus and plus stutters were measured as a percentage, derived by dividing observed stutter peak height by the observed true allele peak heights. Stutter data was used from all genotypes, regardless of spacing of true heterozygote alleles. Analysis for these studies was carried out on 1092 population samples run on an Applied Biosystems 3500xL Genetic Analyzer using a 150 RFU peak height minimum threshold (minimum stutter peak height threshold of 20 RFU). The data in this section were generated with an early, pre-reformulation version of GlobalFiler™ Master Mix (see above section: PCR Primer Set and Master Mix components). A subsequent study using DNA from the same donor stocks and conducted with the final formulation master mix was conducted to demonstrate functional equivalence of the two master mixes in this type of study (data not shown).

### Population and concordance studies

The genotypes of 1194 individuals from samples representing four major populations in the U.S (African American (330), Caucasian (343), Hispanic (368) and Asian (153)) were determined using the GlobalFiler™ Kit under standard conditions. All samples were from purchased stocks from the Interstate Blood Bank (Memphis, TN) or Boca Biolistics (Coconut Creek, FL) described. The donor samples were collected in the United States from unrelated individuals of self-reported ethnicities. GlobalFiler™ genotype concordance was compared to previously typed genotypes generated using the Identifiler™ Plus and NGM SElect™ Kits. (Note: of all 1194 donor samples successfully run with the GlobalFiler™ Kit, one donor sample run for Identifiler™ Plus Kit and 11 donor sample runs for for NGM SElect™ Kit were disqualified due to amplification failures and/or injection run failures.) Allele frequencies and Probability of Identity (PI) values for the GlobalFiler™ Kit loci were determined as described [20]. The data in this section were also generated with an early version of GlobalFiler™ Master Mix (see section above), which did not impact allele calling of the samples [13].

An Amelogenin-Y null male sample (IBB-960) originating from a self-reported Hispanic male donor was identified and profiles generated for this individual with GlobalFiler™ and Identifiler™ Plus Kits were compared. (Gender was independently verified with the Yfiler Plus kit. This individual has a large internal deletion on the Y-chromosome spanning not only the amelogenin Y gene but multiple Y-STRs (DYS570, DYS576, DYS458, DYS449, DYS481 and DYS627)) [21].

### Color balance

Color balance was calculated from the population study done to validate stutter values mentioned in the Stutter section above. DNA was from the same donor stocks described in Population and concordance section above. Actual DNA concentrations for these donor DNA stocks may vary somewhat from nominal concentration due to factors such as minor degradation and/or buffer evaporation over time. Samples in this analysis that showed either off-scale peaks or an average peak-height per sample value under 1000 RFU, indicating sub-optimally high or low DNA input, were excluded, as DNA inputs well above or below the standard recommended amount can adversely affect color balance.

### Statistical analysis and color balance definitions

The terms heterozygote peak height ratios and intra-locus balance (ILB) are equivalent and used interchangeably in this report. These values were calculated for a given locus by dividing the lower allele peak height of a heterozygous locus by the higher allele peak height, with the result expressed as a percentage. Similarly, intra-color peak height ratios and intra-color balance (ICB) are equivalent terms and are used interchangeably in reference to a value calculated in the following manner: first, all heterozygous peaks are averaged and homozygous peaks are halved; once normalized in this manner for diploidy, the lowest value (height) observed among all loci in a given dye channel is then divided by the highest value (height) observed in that dye channel, with results reported as a percentage. Y-based loci, DYS391 and Y_indel_, are not common to all donors and thus were not included. Statistical analyses were performed using Minitab™ or JMP™ software.

## Results

### PCR-based conditions

#### Cycle number

**Fig. 1:**
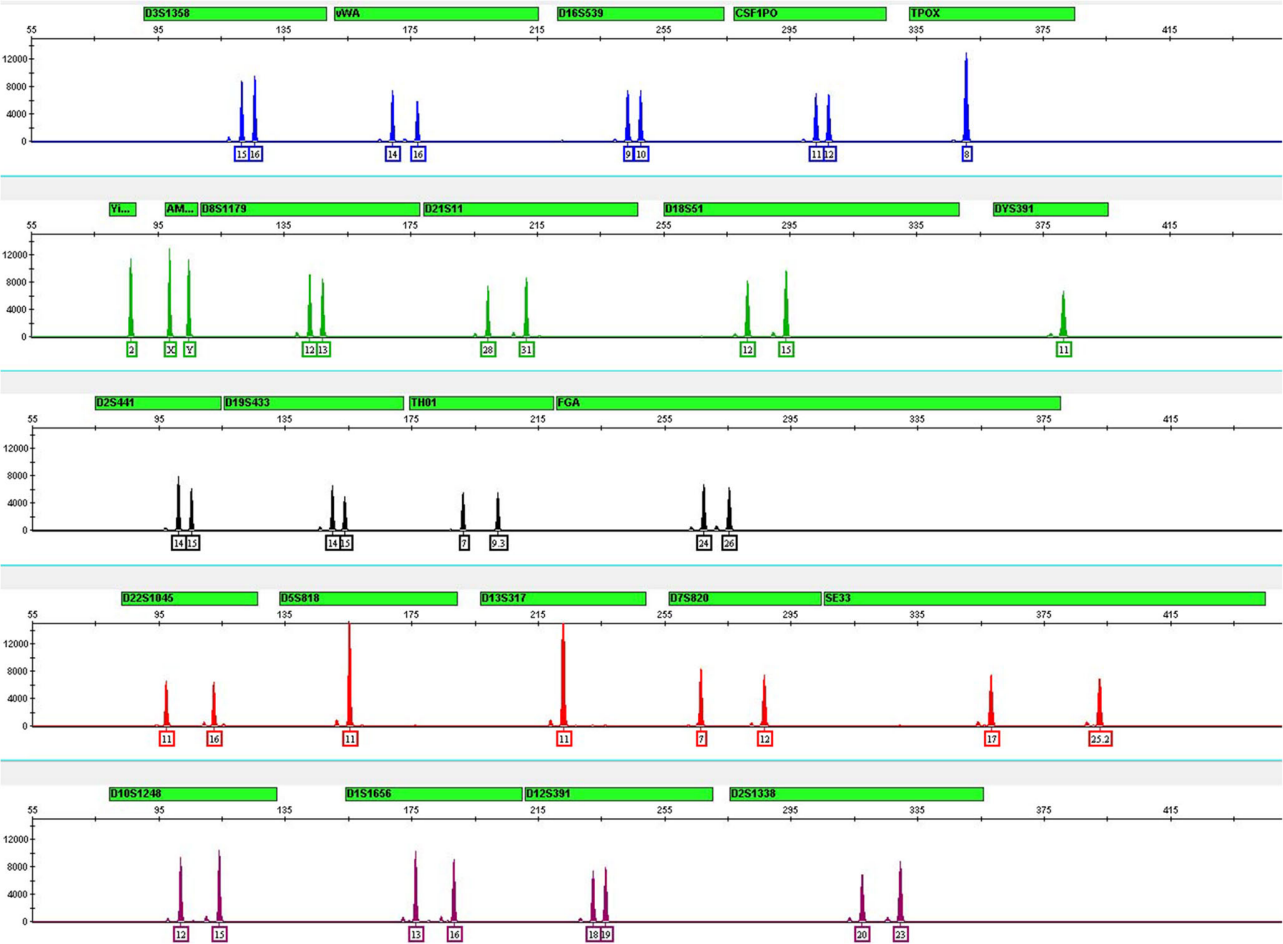
Control DNA 007 profile with standard thermal cycling conditions. Representative profile generated from 1.0 ng of Control DNA 007 amplified with GlobalFiler™ Kit for 29 cycles and electrophorese on an Applied Biosystems 3130xl Genetic Analyzer

The GlobalFiler™ Kit has been optimized for amplification of 1 ng of total input DNA and reactions over a range of amplification cycle numbers (27 to 31) have been examined. As expected, an increase in cycle numbers increased overall peak heights, with some degree of detector saturation due to excess signal intensity (off-scale peaks) observed at 30 and 31 cycles. 29 cycles was determined to be optimal, with respect to maximizing assay sensitivity while minimizing the possible occurrence of off-scale peaks (see Fig. 1).

#### Annealing step

**Fig. 2:**
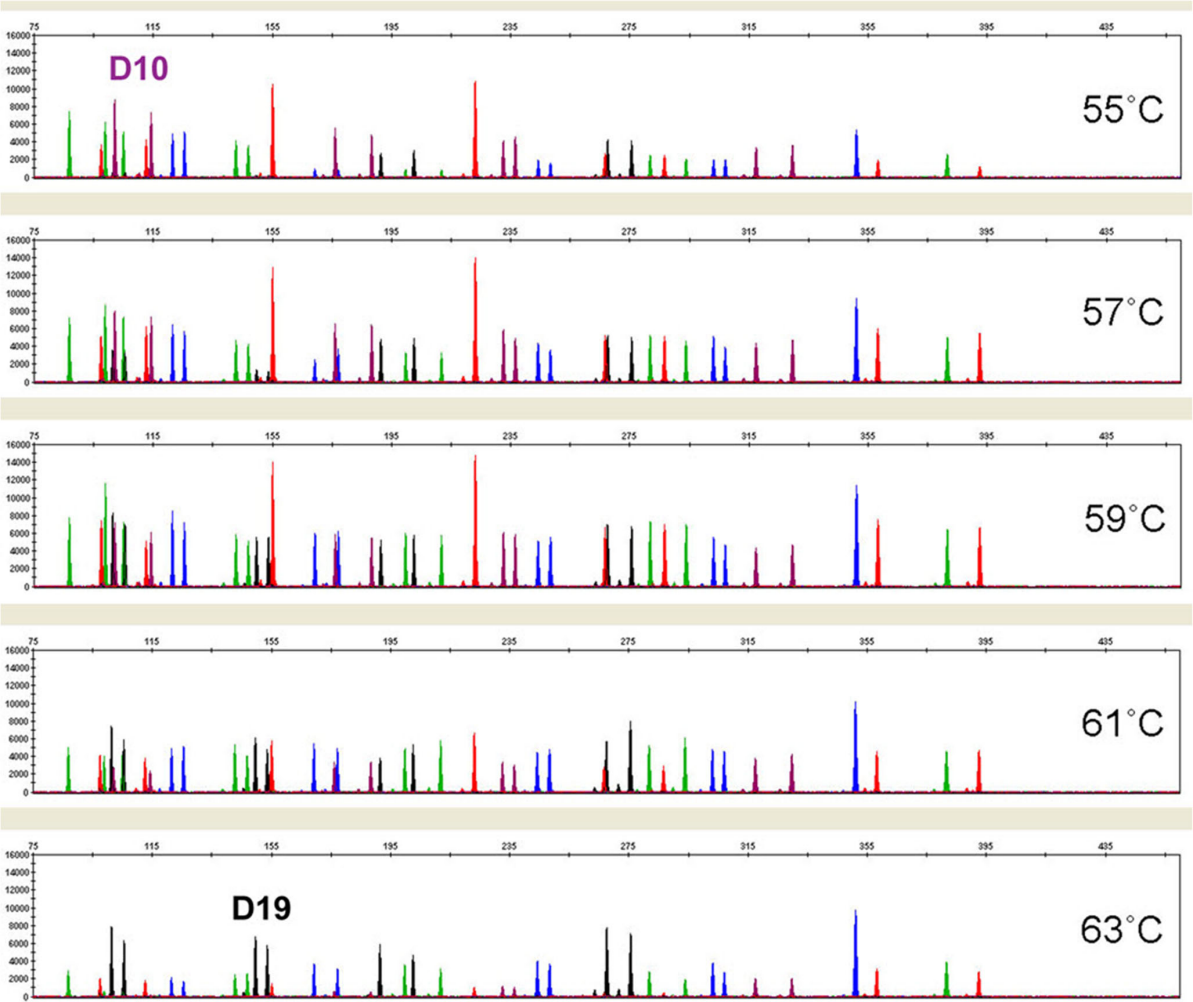
Annealing/extension temperature analysis. 1.0 ng of Control DNA 007 was amplified with GlobalFiler™ Kit for 29 cycles over indicated range of annealing/extension temperatures. Markers D10 and D19 were the most profoundly impacted loci. Reaction products were analyzed on an Applied Biosystems 3500xL Genetic Analyzer (Y-axis scale 0 to 16,000 RFU)

GlobalFiler™ thermal cycling conditions were tested for robustness by varying important parameters such as denaturing temperature, annealing/extension temperature, and final extension time. Standard recommended thermal cycling conditions for the GlobalFiler™ Kit include a 95 °C/1 min activation step; a 94 °C/10 s denaturing step (29 cycles); a 59 °C/90 s annealing/extension step (29 cycles); and a 60 °C/10 min final extension step. Once optimal conditions were identified, the robustness of the GlobalFiler™ Kit was examined by varying the times and temperatures over a relevant range in reactions using 1.0 ng of Control DNA 007. In amplifications where the denaturing temperature was varied by +/− 1.5 °C, profiles returned showed no apparent effect on performance (data not shown). For the annealing/extension step, temperatures of 55, 57, 59, 61, and 63 °C were tested. Here, low temperatures caused a drop in overall peak heights, with the D19S443 locus in the NED™ channel being most affected (see Fig. 2). High annealing/extension temperatures also suppressed overall peak height levels, in this case, with D10S1248 in the SID™ channel being the most affected locus. Intra-color balance decreased when the annealing/extension temperature deviated from the optimal temperature of 59 °C by more than 2 °C. Annealing/extension temperatures 2 °C above or below optimum did not affect the GlobalFiler™ Kit’s ability to generate full STR profiles.

#### Final extension step

**Fig. 3:**
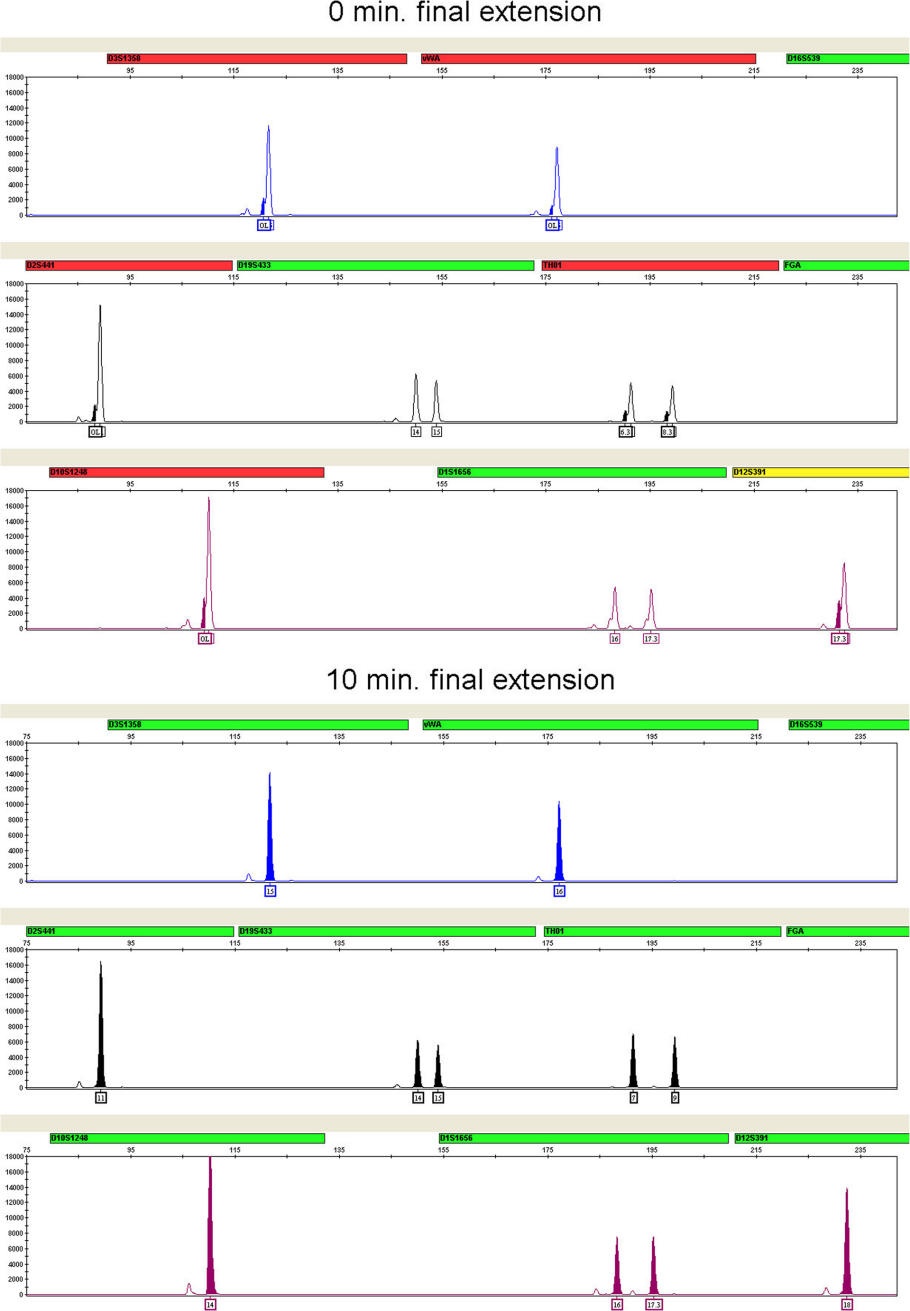
Omitting the final extension step results in shoulders on main allele peaks due to incomplete A nucleotide addition. Final extension step times of 0 min (top three panels) and 10 min (bottom three panels) were tested. Examples shown are the smaller amplicons of 6-FAM™, NED™, and SID™ dye channel data from a 3500xL Genetic Analyzer using the GlobalFiler™ Kit

Non-templated terminal nucleotide addition is an activity intrinsic to the type of DNA polymerase used in the GlobalFiler™ Kit [22]. This addition, typically of adenosine, results in a PCR product that is one nucleotide longer than the predicted amplicon, often referred to as the “+A” form. In STR multiplexes, a post-amplification final extension step is commonly included to ensure that nucleotide addition goes to completion on all amplified products in order to maintain uniform electrophoretic sizing. Final extension times of 0, 5, 10, 15, and 20 min at 60 °C were evaluated. Incomplete +A addition is most readily detected by the occurrence of “split” or “shoulder” peak morphologies that can give rise to spurious off-ladder or micro-variant “allele peaks” being called. Incomplete +A addition effects were clearly evident with final extension times of 0 and 5 min in our experiments. Complete +A addition was observed after 10 min of incubation time (see Fig. 3). No performance benefits were seen with increased extension times greater than 10 min and no other significant effects on performance were seen with any of the extension times tested.

#### PCR reaction components

**Fig. 4:**
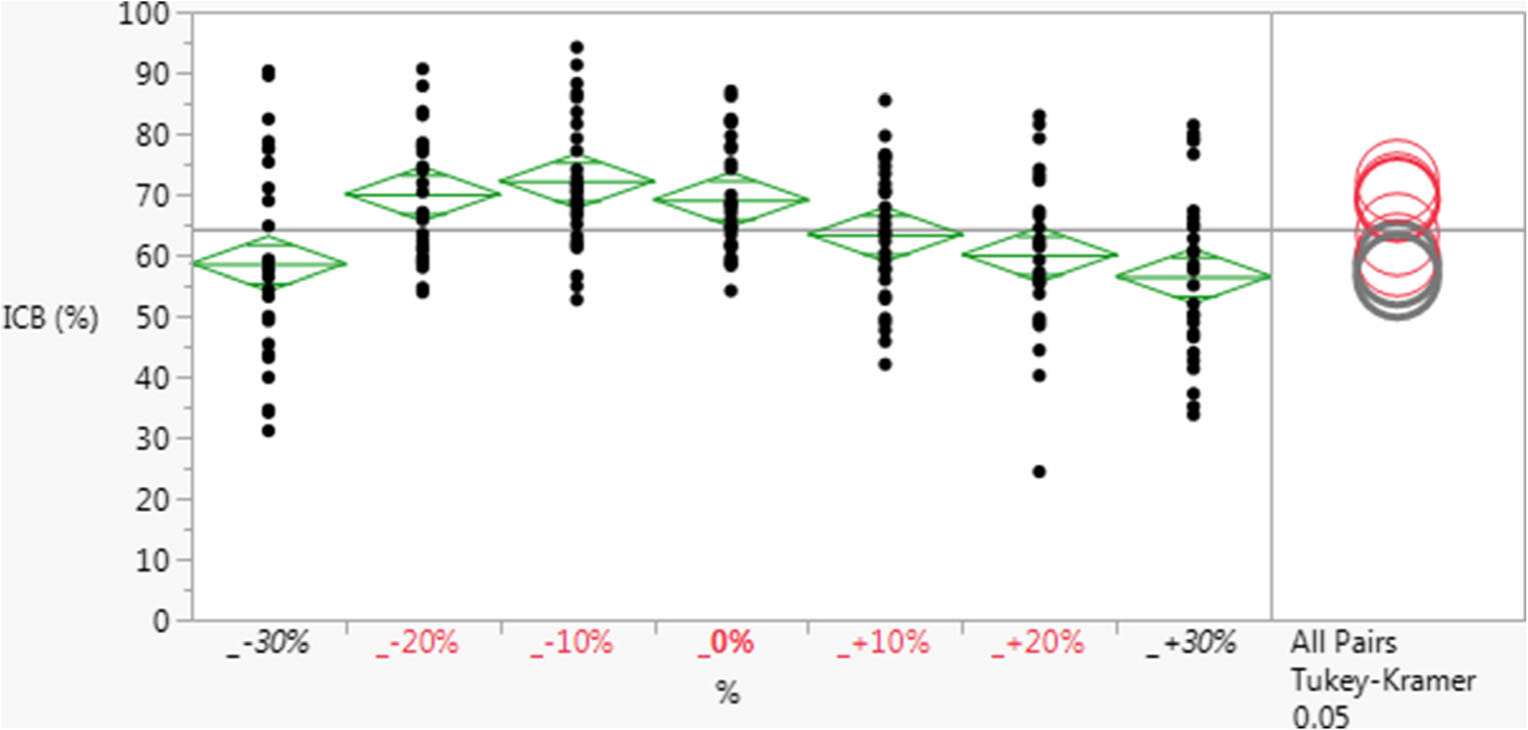
GlobalFiler™ Master Mix with varied magnesium concentrations. Shown are the average intra-color balance (ICB) ratios over all dye channels expressed as percentages) observed in profiles returned from reactions (*n* = 3 replicates for each condition) in which the total concentration of magnesium in the reaction was varied +/− 30% of standard concentration (“0%”), in 10% increments. One way comparison of means by All Pairs Tukey-Kramer method shows statistically significant lower average ICB values only when magnesium concentration is 30% more or less than standard concentration (0%)

GlobalFiler™ Primer Set and Master Mix components were optimized through a design of experiments approach to provide for maximal sample input volume, minimal reaction time, maximal inhibitor tolerance, and maximal sensitivity with minimal background or spurious peaks. Once optimal conditions were identified, the robustness of the GlobalFiler™ assay was examined by guard-band testing in which (a) raw material concentrations in the Master Mix, or (b) reagent input volumes in reactions, were varied over a range of minus to plus 30% (*v*/*v*), in 10% increments. As a performance metric, profiles were analyzed for average intra-color balance (ICB) ratios over all dye channels, with values being expressed as percentages. As seen in Fig. 4, the Master Mix was robust (no significant change in ICB) with as low as 80% and as high as 120% of standard concentration of magnesium (*p* ≤ 0.05). With respect to varied Master Mix or Primer Set reagent input volumes (also spanning +/− 30% normal) in reactions, the assay was also robust over a similar percentage range of input amounts, by ICB; and full profiles were returned under all conditions, with the exception of runs with 70% normal Master Mix input volume (data not shown).

Similar studies were conducted on other individual Master Mix components and select combinations of Primer Sets (data not shown). Magnesium concentration levels in the Master Mix were highly and positively correlated with peak height levels and were optimized to deliver maximal assay sensitivity and color balance in combination with minimal stutter and background generation (data not shown).

### Species specificity

**Fig. 5:**
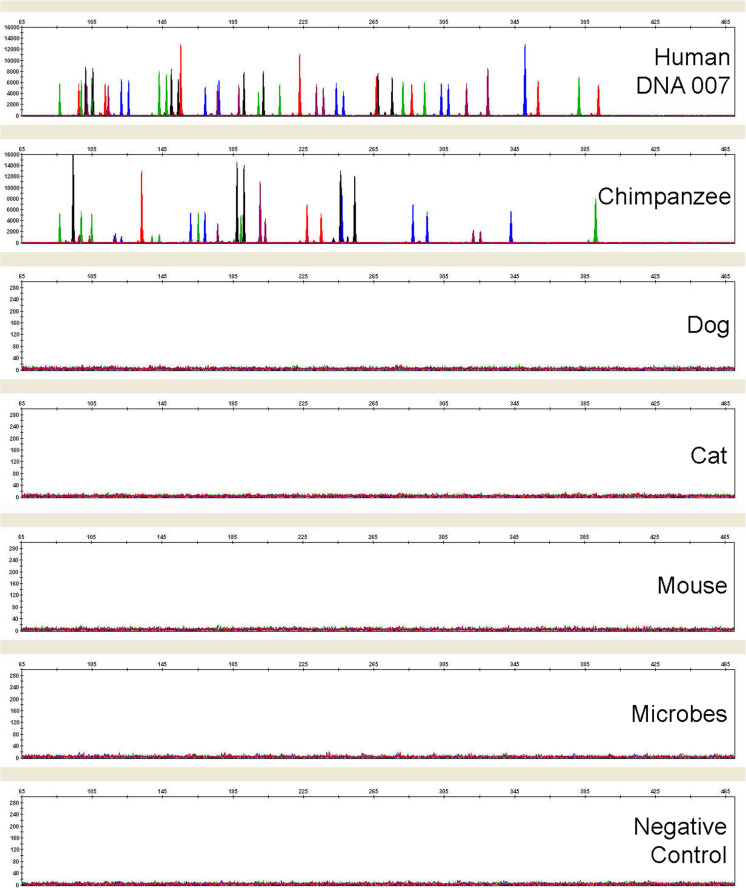
Representative electropherograms from a study examining GlobalFiler™ Kit specificity toward human genomic DNA versus non-human DNA samples from: a primate (1 ng, chimpanzee), non-primate animals (10 ng each), and pooled microorganisms. Substantial cross-reactivity is seen only in the primate sample. All electrophoresis was conducted on the same 3500xL Genetic Analyzer

As expected, the GlobalFiler™ Kit is cross-reactive with primate DNAs from chimpanzee (see Fig. 5) and other non-human primates (data not show). However, almost all of the cross-reactivity peaks in these cases were designated as “Off-Ladder” since they did not fall into the bins of their respective loci location (data not shown). Several non-primate animal and microbial DNAs were also tested and most produced no peaks over a threshold of 175 RFU (all electrophoresis performed on the same 3500xL instrument). Minor cross-reactivity was seen with horse, pig and chicken DNA at 94 bp (VIC™ channel, less than 1800 RFU, due to Amelogenin cross-reactivity), 424 bp (TAZ™ channel, less than 1200 RFU) and 328 bp (TAZ™ channel, less than 200 RFU), respectively. More detailed information on species specificity can be found in the User Guide [13].

### Inhibition models

**Fig. 6:**
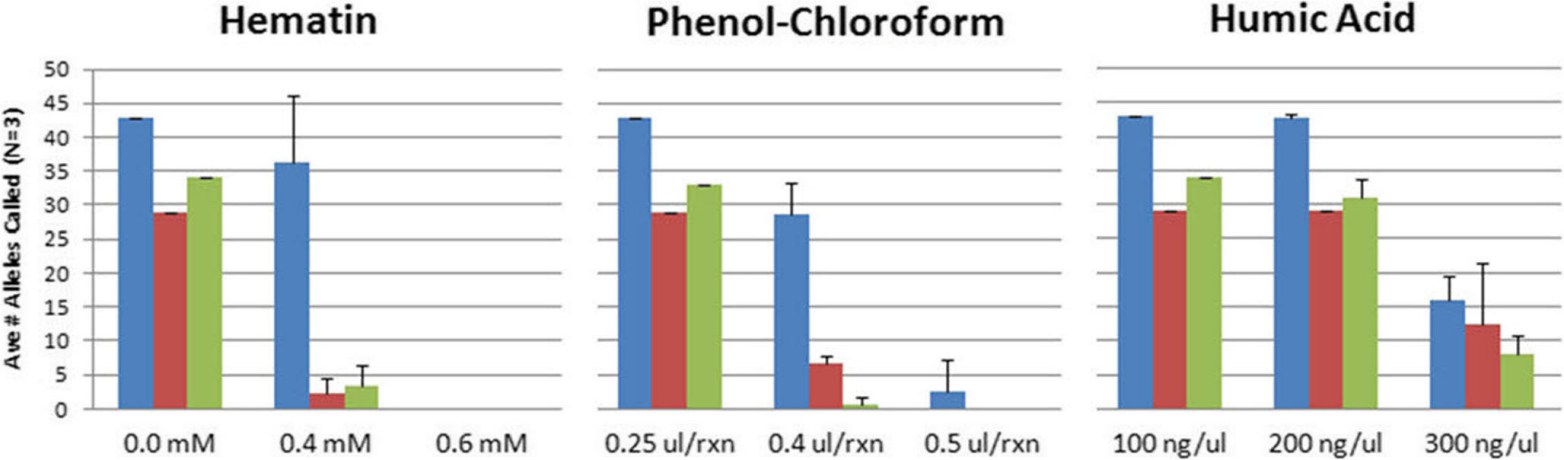
Using standard protocol, 1.0 ng Control DNA 007 was run with the GlobalFiler™ (blue), Identifiler™ Plus (red) or NGM SElect™ (green) Kits in the presence or absence of inhibitory substances indicated, at concentrations denoted along x-axes. The average number of alleles correctly recovered in triplicate amplifications (*n* = 3) is presented. The number of alleles in a full profile from uninhibited runs (see Hematin plot) for GlobalFiler™, Identifiler™ Plus and NGM SElect™ Kits is 43, 29 and 34, respectively. In all cases, with increasing levels of inhibition, GlobalFiler™ Kit returned either an equal or significantly higher number of allele calls

Substances known to be inhibitory toward PCR reactions are often encountered in DNA extracted and isolated from forensic casework samples with typical downstream effects observed consisting of loss of peaks and/or lowering of peak heights in profiles generated. These inhibitory agents may originate from carry-through of small amounts of the upstream extraction chemistries (such as phenol chloroform), or from elements intrinsic to the sample itself (such as hematin from blood and humic acid from soil) [18, 19]. To model such scenarios and test the stability and robustness of the GlobalFiler™ assay in this context, we conducted a study in which increasing amounts of such inhibitory substances were added directly to the amplification reactions along with 1 ng of Control DNA 007. Performance levels were assessed based on the number of alleles correctly called in profiles returned from these reactions. As a performance benchmark, NGM SElect™ and Identifiler™ Plus Kits were run in parallel in this study. The GlobalFiler™ Kit returned profiles with a significantly higher number of allele calls than either of the other kits in the presence of 0.4 mM hematin and 0.4 μL per reaction of phenol chloroform (Fig. 6). These data demonstrate that, in comparison to existing Thermo Fisher Scientific STR chemistries, the GlobalFiler™ Kit can generate an equal or greater number of alleles in the presence of high inhibitory substances. Information on average peak heights observed and recovery of alleles common to GlobalFiler™ and legacy kits in this study can be found in Online Resource 5.

### Sensitivity

**Fig. 7:**
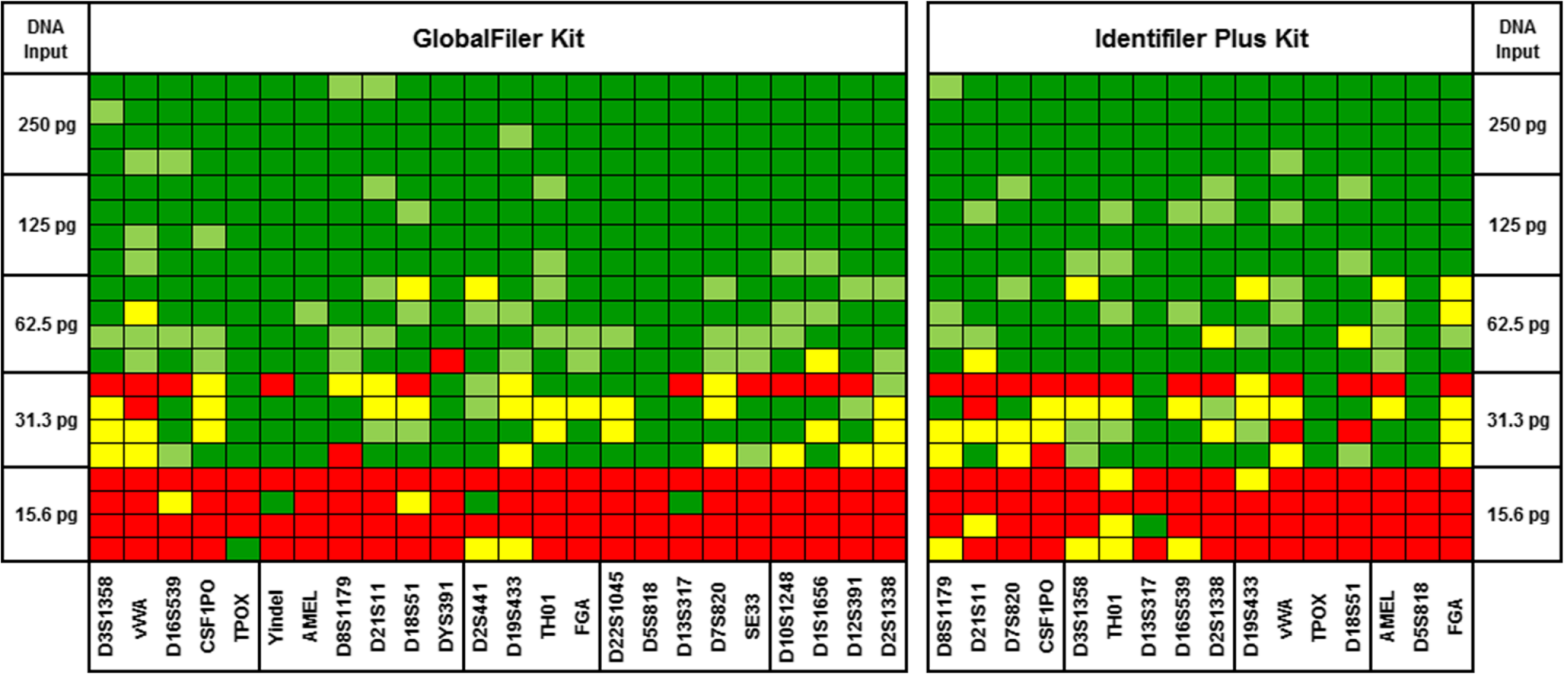
Assay sensitivity. Using standard protocols, four replicate (*n* = 4) of GlobalFiler™ (left) and Identifiler™ Plus (right) Kit reactions were run over a range of Control DNA 007 input amounts (total per reaction) as denoted on the Y-axis. Results for each locus are presented in the heat map. Green indicates allele(s) properly called (lighter green shading indicates heterozygote peak height ratio observed was below 60%), red indicates no allele(s) called, and yellow indicates one of two heterozygote alleles was called. Loci sharing the same dye channel are boxed, and are ordered therein according to size, beginning with the smallest

Sensitivity of the GlobalFiler™ Kit was tested using a range of DNA inputs, with the Identifiler™ Plus Kit included in this study for benchmarking purposes. Amplified products from all reactions were analyzed on the same 3500xL instrument to control for potential instrument-to-instrument variability in signal intensity. The total number of correct alleles called was noted over four replicates for both kits. Full profiles were obtained consistently at 125 pg input or greater with both chemistries (Fig. 7). At 15.6 pg, less than 3 alleles on average were returned by either kit. In addition to serial dilutions of template DNA, non-template controls (NTCs) were also run. No background noise or peaks of any kind were observed above 30 RFU in the read region for any of the four replicates (see Online Resource 3). To test the effects of high (above optimal) template DNA input on GlobalFiler™ performance, runs were also performed with 007 DNA input of 1 ng (control), 2 ng and 3 ng. Relative to the control, in profiles from runs with three times the standard DNA input (3 ng), peak heights were elevated, as expected, with some low background peaks clearly emerging; however, these artifact peaks were largely pull-up peaks that are clearly distinguishable from the true allele peaks, with the profiles also remaining fairly well balanced (see Online Resource 4).

### Degraded DNA

**Fig. 8:**
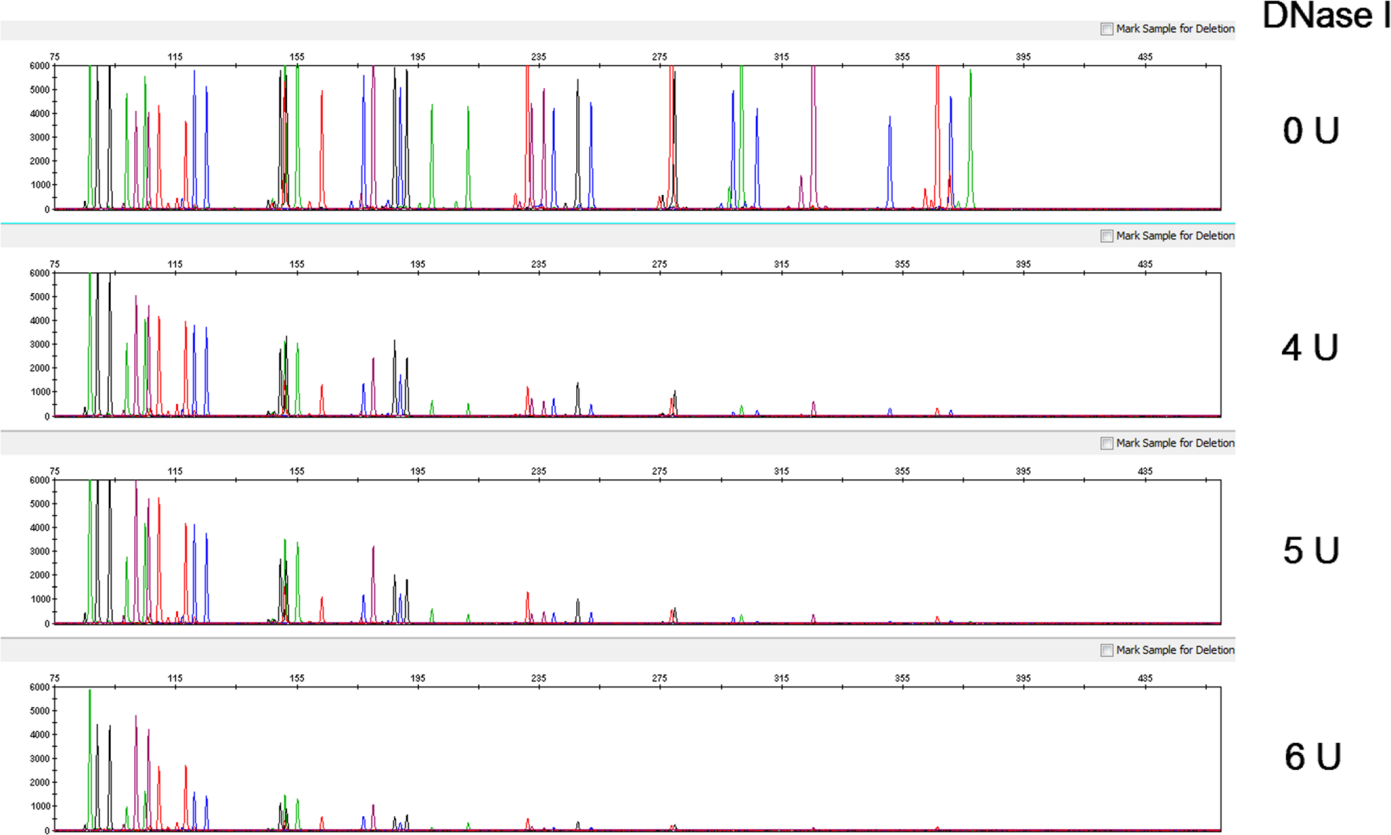
Degraded DNA study electropherograms. Representative profiles from GlobalFiler™ reactions using 1.0 ng of control genomic DNA (Raji) that had been subjected to controlled degradation. Collectively, the profiles show a trend of diminished recovery of larger alleles with increasing levels of degradation. Y-axis scale equals 6000 RFU

It is well-known that degradation of sample DNA due to exposure to environmental factors can result in a reduction in the number of intact template DNA in size ranges necessary for the successful PCR amplification of STR multiplex loci of interest. To examine such potential effects on the GlobalFiler™ Kit performance under controlled conditions, degraded DNA was experimentally generated and amplified under otherwise standard conditions. For this model, high-molecular-weight control DNA was subject to graded degradation with increasing amounts of DNase I enzyme (0, 4, 5, and 6 Units) before being amplified with the GlobalFiler™ Kit [23]. Increasing preferential loss of larger loci was observed in profiles from DNA with increasing amounts of degradation (Fig. 8). These results are consistent with a model in which smaller STR targets are less vulnerable to DNA degradation. As expected, the degraded DNA profiles characteristically consisted of small amplicons exhibiting lower signal intensity. With the most highly degraded DNA (6 Units), an average of approximately 26 alleles were still returned in three replicate runs, and, under all conditions in this study, the GlobalFiler™ Kit returned more alleles than Identifiler™ Plus (see Table 1 and Online Resource 6). With highly degraded DNA, smaller loci were preferentially amplified in each dye channel by both kits in a similar manner. Under such conditions, the GlobalFiler™ Kit appeared to benefit primarily from having more loci targets in its multiplex and an additional dye channel within which amplify targets.

**Table 1:** Degraded DNA study allele counts. Total number of alleles returned from 3 replicate GlobalFiler™ Kit and Identifiler™ Plus Kit reactions using 1.0 ng of control genomic DNA (Raji) that had been subjected to controlled degradation

DNase I	Control			4 U			5 U			6 U		
GlobalFiler	40	40	40	37	37	38	39	35	36	27	25	27
Identifier Plus	28	28	28	28	27	28	28	27	26	21	22	20

### DNA mixture studies

**Fig. 9:**
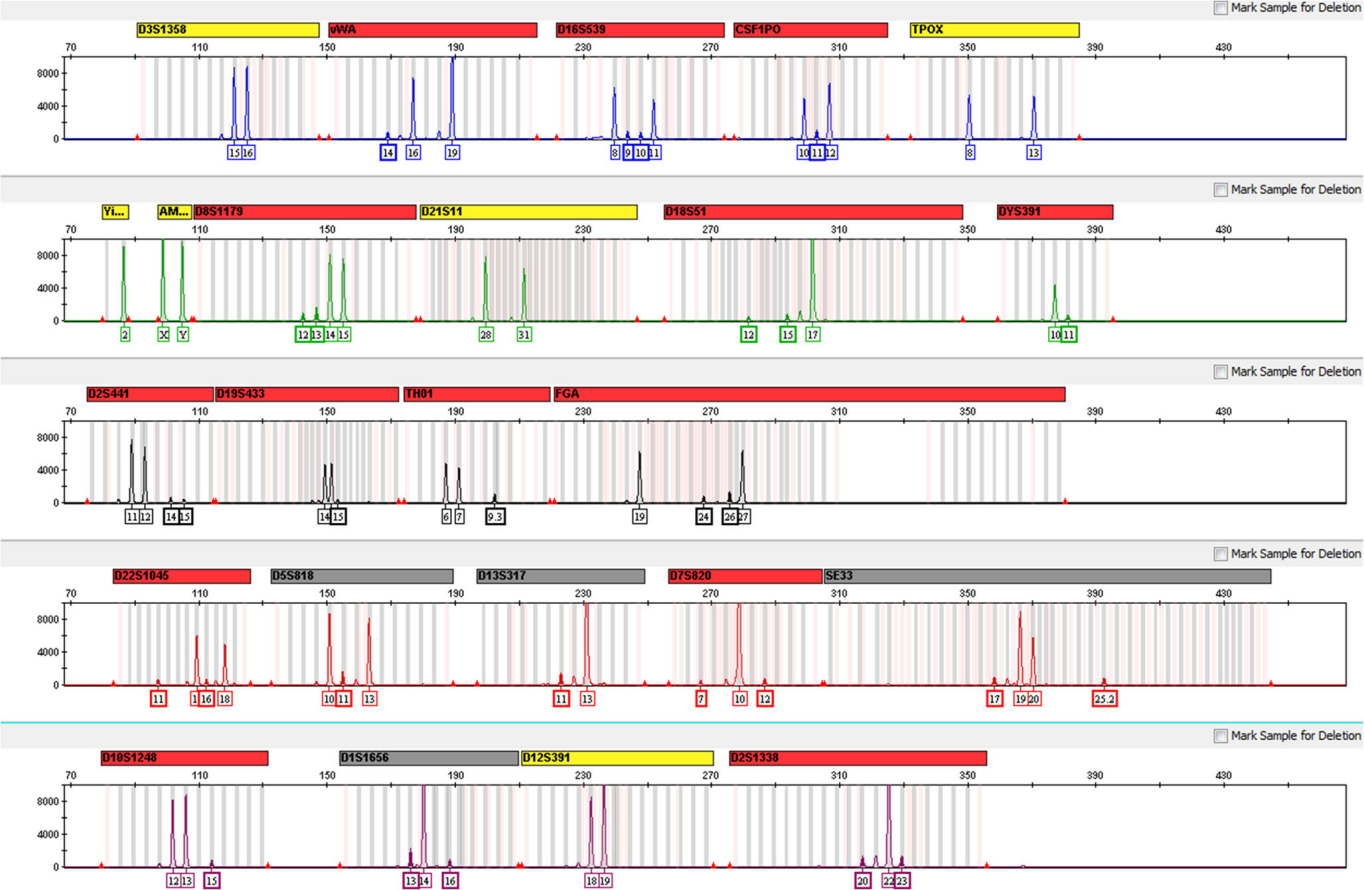
Mixture study electropherogram. Electropherogram depicting the recovery of all non-overlapping minor alleles in the 8:1 Raji:007 DNA mixture. The minor contributor 007 DNA (111 pg input DNA) alleles are highlighted

Mixtures of DNA originating from more than one individual are frequently encountered in forensic casework samples. As such, the ability of any STR multiplex assay to distinguish between minor and major contributor DNA is of great importance. To model a forensic mixture sample and test the performance of the GlobalFiler™ Kit in this context, two control DNAs, Raji and 007 DNA, were used (see Online Resourse 1, Mixture Study genotypes). These two DNAs were combined in various ratios (0:1, 1:1, 5:1, 8:1, 1:0) with diminishing relative amounts from one (007 DNA) representing the “minor component”. The total amount of combined input DNA at each ratio was held constant at 1 ng for all reactions. An electropherogram with profiles from 1:8 ratio mixture and minor contributor peaks highlighted is shown in Fig. 9. As seen in Table 2, detection of full profiles (excluding overlapping alleles) for the minor contributor was achieved in six of six runs at the 1:5 ratio input (167 pg of minor component DNA present) and in three of six runs at the 1:8 ratio input (111 pg of minor component DNA present). In all three incomplete minor contributor profiles in the last set, the missing peak was allele 13 at D1S1656.

**Table 2:**
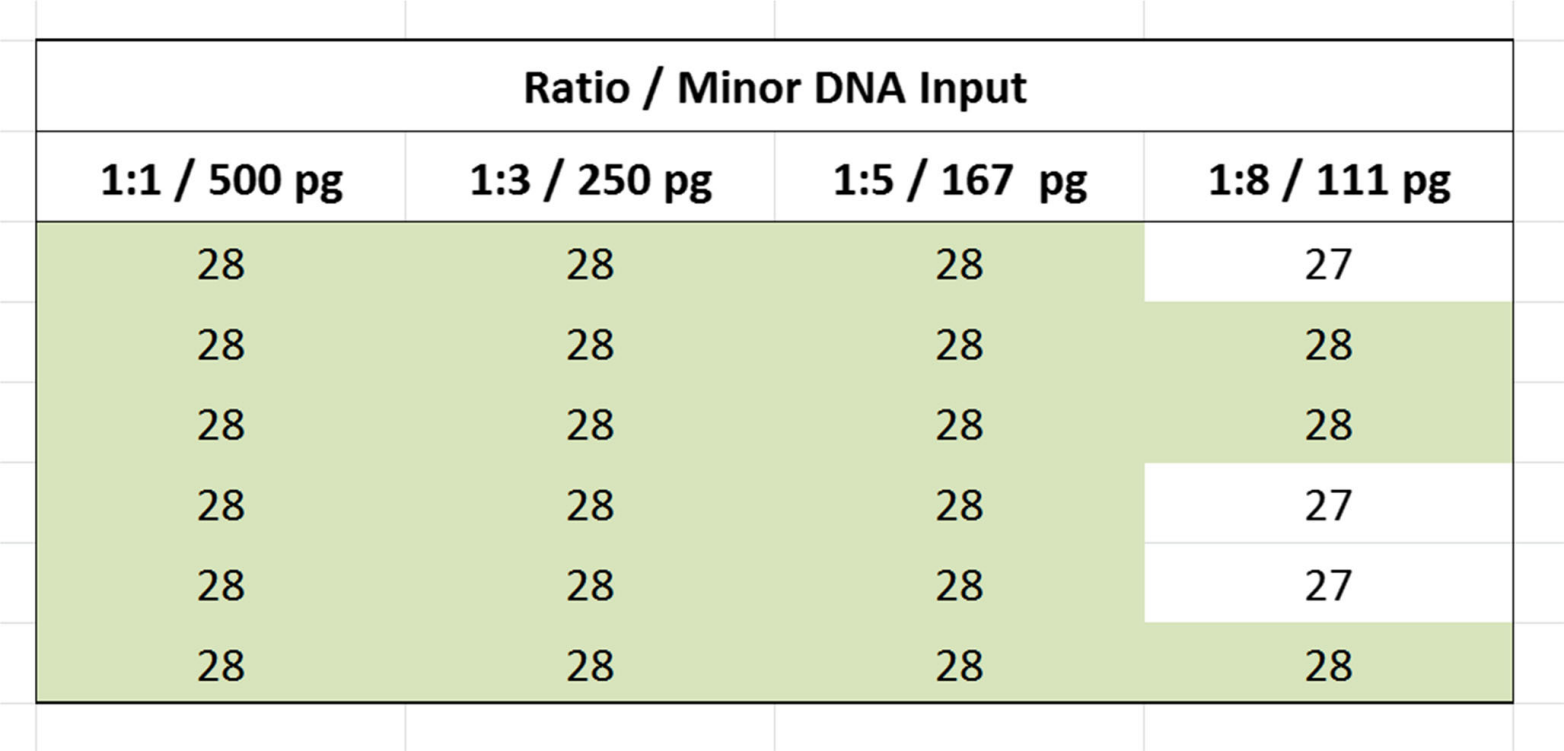
Mixture study. Number of non-overlapping minor contributor alleles recovered (total possible is 28 and is indicated by green shading) with donor input ratios ranging from 1:1 (500 pg of minor contributor DNA present in run) to 1:8 (111 pg of minor contributor DNA present in run) and total input DNA held constant at 1 ng (*N* = 6)

### Bone sample

**Fig. 10:**
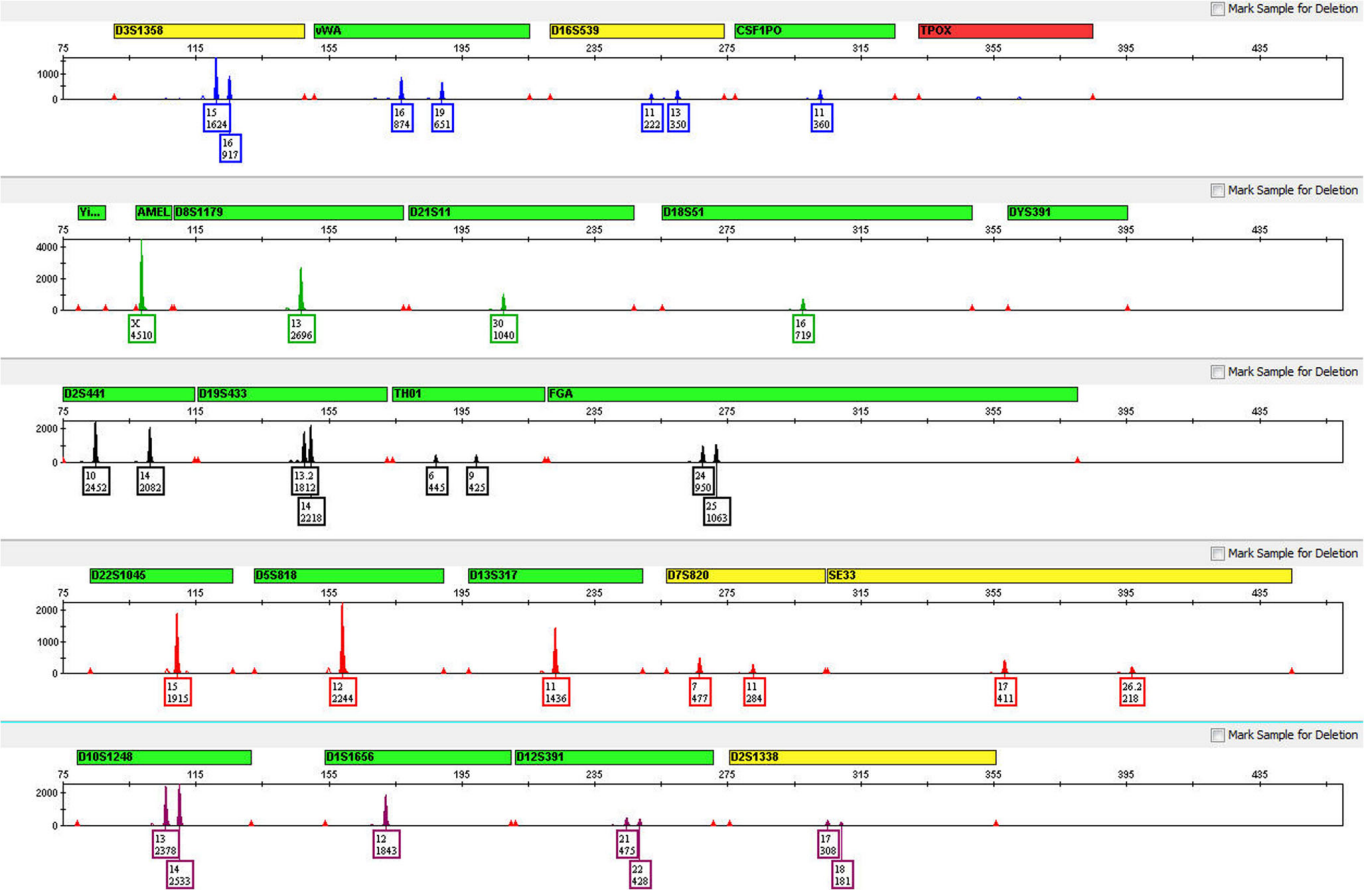
Bone sample analysis. Representative profile returned by GlobalFiler™ Kit from 1.0 ng of DNA derived from a challenging bone specimen known to possess degraded DNA. Alleles were captured in 21 of the 22 autosomal GlobalFiler™ markers in this replicate run. (Note that Y-axes are scaled independently for each dye channel to maximally capture dynamic range of peak heights)

Particularly challenging casework samples are often encountered in the form of bone or teeth tissue, which, in addition to potentially possessing inhibitory factors, tend to yield limited DNA that has been subject to environmental exposure, and thus typically some degree of degradation. We examined the performance of the GlobalFiler™ Kit and that of two other commercially kits on mock casework sample DNA that had been isolated from a bone specimen known to possess DNA of compromised quality. By real-time PCR quantification, which targets relatively small loci, the concentration of DNA present in the prepared sample was determined sufficient for an input of 1 ng total DNA in STR reactions for all kits. In replicate reactions, the GlobalFiler™ Kit returned an average of 32.5 alleles (93% of total possible) from this sample input, while Identifiler™ Plus and NGM SElect™ Kits both returned an average of 23.5 and 28, respectively (94% and 100% of total possible, respectively). Figure 10 shows a representative profile from this sample returned by GlobalFiler™. This study demonstrates the ability of the GlobalFiler™ Kit to return a higher number of allele calls from highly challenging casework-type samples than other commercially available kits. Information on the percentage of shared alleles recovered and average peak heights observed with this sample can be found in Online Resource 5.

### Mock casework samples

DNA from mock casework samples prepared in-house (and described in Methods Section) was analyzed with GlobalFiler™ and Identifiler™ Plus Kits. Over this sample set, the average number of alleles returned per sample by GlobalFiler™ Kit was 35.0 compared to 23.5 alleles for Identifiler™ Plus Kit (see Table 3). Differences in DNA inputs are attributed to a combination of low DNA concentration in certain samples and different maximum sample volume inputs capabilities of 15uL and 10uL, for the GlobalFiler™ and Identifiler™ Plus Kits, respectively.

**Table 3:** Mock casework samples. Alleles recovered from mock samples (described in Methods Section) in GlobalFiler™ (GF) and Identifiler™ Plus (IDP) runs were tabulated. Percentage of alleles recovered out of total possible (when known) for each kit are also presented. Quantification values for the extracted samples are also presented, along with DNA input amounts for the STR amplification reactions

Sample	Sample DNA	DNA input (ng/reaction)		Alleleles called		% of tot. alleles	
	(ng/μL)	GF	IDP	GF	IDP	GF	IDP
Soda Can Swab 1	0.081	1.00	0.81	40	26	100%	100%
Soda Can Swab 2	0.185	1.00	1.00	40	26	100%	100%
Soda Can Swab 3	0.079	1.00	0.79	40	26	100%	100%
Cig Butt 1	0.012	0.19	0.12	18	11	n/a	n/a
Cig Butt 2	0.983	1.00	1.00	40	28	n/a	n/a
Cig Butt 3	0.830	1.00	1.00	40	28	n/a	n/a
Blood on Soil Day0	3.419	1.00	1.00	40	27	100%	100%
Blood on Soil Day1	0.757	1.00	1.00	40	27	100%	100%
Blood on Soil Day2	0.004	0.06	0.04	24	18	60%	67%
Blood on Soil Day3	0.001	0.01	0.01	0	0	0%	0%
Blood on Wood	0.014	0.21	0.14	41	27	100%	100%
Blood on Metal	0.025	0.38	0.25	41	27	100%	100%
Blood on Metal (Flocked Swab)	0.018	0.28	0.18	41	27	100%	100%
Semen on Cloth	2.783	1.00	1.00	41	27	100%	100%
Semen on Cloth with UV	0.214	1.00	1.00	41	27	100%	100%
Average per sample				35.0	23.5		

### Stutter

In PCR amplification of STRs, some amount of a minor product that is typically one repeat unit smaller (and much lower in peak height) than the true target allele is always observed. Such products, commonly referred to as “stutter”, have been attributed to slippage at the polymerase/strand interface during strand elongation [24]. This most common form, at n – 1 repeat unit from the true allele, is often referred to as “minus stutter”, in contrast to the much less common form, known as “plus stutter” (or “forward stutter”), which is one repeat unit *larger* than the true allele. Historically and in this assay, the latter is routinely seen with the trimeric marker, D22S1045, and also infrequently in some tetrameric markers, such as SE33. Other, more complex occurrences of stutter are also known and observed in this assay, specifically minus stutter in the form of a peak at n – 2 nucleotides (“1/2” of a repeat unit) from the true allele, as seen with SE33, and another tetrameric marker, D1S1656. As these peaks can confound efforts to identify and interpret true donor peaks, they are routinely characterized as an average percentage value (relative to the adjacent true allele peaks in a large sample set), referred to as “percent stutter”, and accounted for in profile analysis methods. For the GlobalFiler™ Kit, profiles from a population of 1092 donor samples were analyzed and evaluated for stutter. As seen previously, observed stutter percentages in this study tended to increase with increasing allele size (data not shown, see User Guide [13]). Results presented in Table 4 show information on mean stutter percentage values for each loci, including the value commonly used to filter stutter peaks at the profile analysis level: mean stutter percentage plus three standard deviations. A separate verification study on stutter using the same methodology and a slightly smaller subset of samples from this donor pool (*N* = 835) was also performed with the current on-market, updated version of the Master Mix (see Materials and Methods section). This study showed no significant differences in average stutter percentages or derived stutter filter values (mean average value +3 standard deviations) relative to the first study (see Online Resource 2 and User Guide [13]). In a smaller study using Control DNA 007 to evaluate stutter levels observed with the GlobalFiler™ Kit in direct comparison to an existing assay, stutter percentages at most alleles from GlobalFiler™ Kit profiles were equal to or lower than those seen in profiles generated with the Identifiler™ Plus Kit (data not shown).

**Table 4:** Stutter mean averages, ranges, standard deviations (SD), and Mean + 3SD values for the GlobalFiler™ Kit marker set. Sample set was DNA form 1092 donors

Locus	Number of observations (*n*)	Stutter mean (%)	Stutter min and max (%)		SD	Mean + 3 SD
CSF1PO	1359	5.22	1.82	10.25	1.1852	8.77
D10S1248	1512	7.07	2.13	12.58	1.4631	11.46
D12S319	1696	7.50	2.11	15.51	2.0544	13.66
D13S317	1275	4.87	1.49	14.51	1.4412	9.19
D16S539	1400	5.16	2.05	9.76	1.4379	9.48
D18S51	1745	6.94	2.68	15.8	1.8249	12.42
D19S433	1591	6.10	2.36	10.94	1.2924	9.97
D1S1656	1744	7.14	2.9	15	1.6897	12.21
D1S1656 (− 2 nt)	242	1.56	0.66	3.38	0.2972	2.45
D21S11	1652	6.69	2.52	13.95	1.2514	10.45
D22S1045	1372	7.69	1.31	21.46	2.8559	16.26
D22S1045 (+ 3 nt)	1002	4.26	0.85	7.34	0.8103	6.69
D2S1338	1861	7.10	2.41	13.79	1.5435	11.73
D2S441	1397	4.54	1.3	12.18	1.1852	8.10
D3S1358	1481	7.18	2.74	14.5	1.2669	10.98
D5S818	1354	5.54	1.8	9.85	1.2056	9.16
D7S820	1275	4.41	1.44	11.46	1.3023	8.32
D8S1179	1566	5.84	1.96	12.99	1.2519	9.60
DYS391	606	5.17	3.45	8.05	0.7557	7.43
FGA	1691	6.84	2.96	12.89	1.5692	11.55
SE33	1991	8.87	3.22	16.81	1.8728	14.49
SE33 (− 2 nt)	1231	2.75	1.27	4.95	0.4063	3.97
TH01	586	2.37	1.03	4.99	0.6946	4.45
TPOX	679	2.81	1.06	10.21	0.9148	5.55
vWA	1516	6.23	1.97	11.75	1.4982	10.73

### Population studies and genotype concordance

To examine concordance, a population study was conducted in which 1194 samples selected from individuals spanning 4 ethnicities (see below) were examined. Extracted DNA from these donor blood samples was analyzed with the GlobalFiler™ Kit and results were cross-checked for concordance against the legacy Identifiler™ Plus and NGM SElect™ Kits. Full genotype concordance was observed in each comparison.

This large-scale population study data was also the basis for determining individual allele frequencies and Probability of Identity (P_I_) for GlobalFiler™ among African American (*n* = 330 samples), Caucasian (*n* = 343 samples), Asian (153) and Hispanic (*n* = 368 samples) donors [20]. (P_I_) values derived for groups shared between this and similar studies for earlier kits are presented in Table 5. Additional data on Asian donors and individual allele frequencies (including Y markers) for all groups can be found in the GlobalFiler™ Kit User Guide [13].

**Table 5:** Probability of Identity. Combined Probability of Identity (PI) values for the GlobalFiler™ Kit loci and existing kits. The genotypes of 1041 individuals, with approximately equal representation among African American (330), Caucasian (343) and Hispanic (368) donors, were determined using the GlobalFiler™ Kit; and observed allele frequencies within each ethnic group were then used to calculate the combined (PI) values. The PI value is the probability that two individuals selected at random will have an identical genotype. Y-specific allele frequencies for GlobalFiler™ Kit loci DYS391 and Y indel were not included in the probability of identity calculation here and can be found in the User Guide [13]

Kit	No. of STR loci	African-American	Caucasian	Hispanic
Identifier Plus	15	1.31 × 10^−18^	5.00 × 10^−18^	7.65 × 10^−18^
NGM	15	6.52 × 10^−20^	2.78 × 10^−19^	2.59 × 10–^19^
NGM SElect	16	8.12 × 10^−22^	2.35 × 10^−21^	3.16 × 10^−21^
Globalfiler	21*	6.18 × 10^−27^	3.71 × 10^−26^	3.09 × 10^−26^

*Autosomal STRs only

**Fig. 11:**
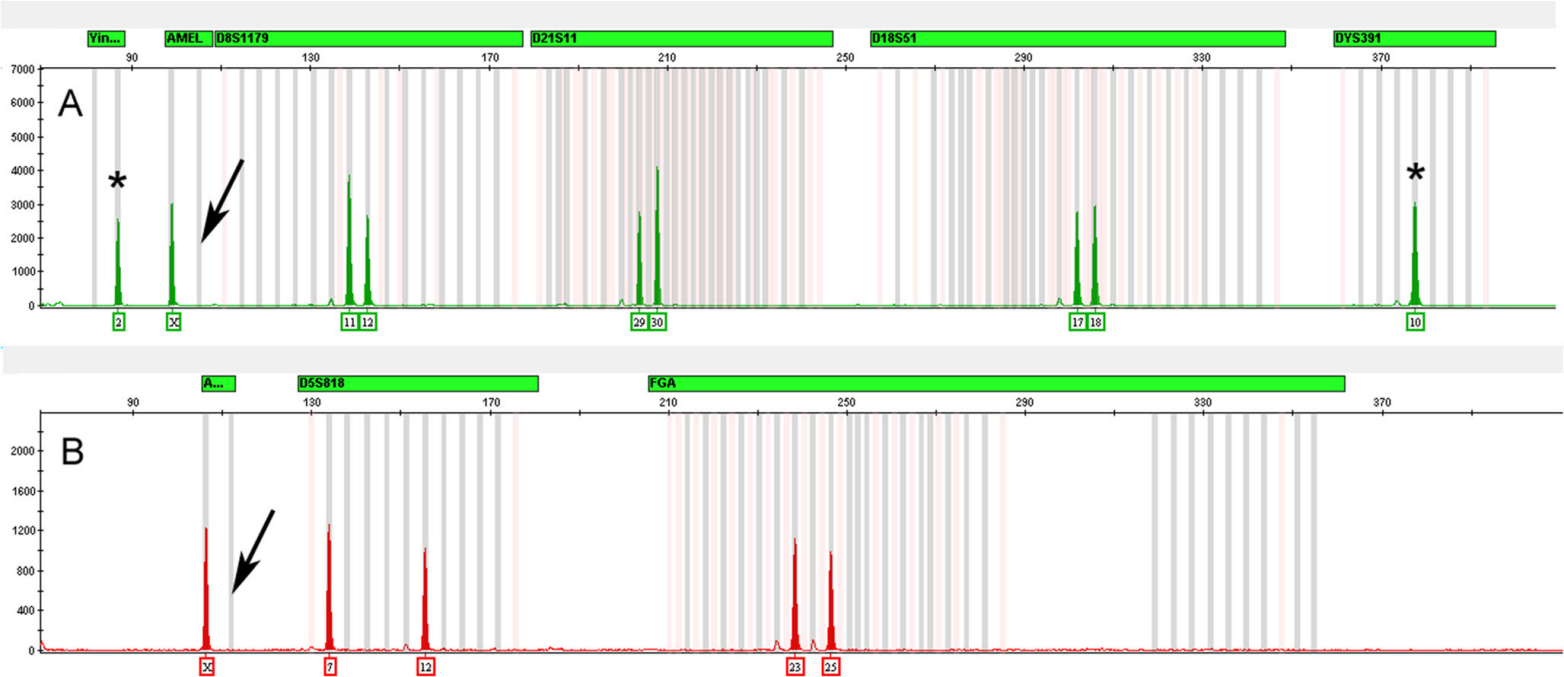
Electropherograms of amplified DNA from an Amelogenin-Y null male. Panel **a** and **b** show the profiles from the Globlafiler and Identifiler Plus kits, respectively. Both profiles show the loss of the Amelogenin-Y allele as shown by the arrows. The asterisks correspond to the Yindel-2 and DYS391-10 alleles in the Globlafiler kit profile

In rare cases, the absence of the Amelogenin-Y allele from an unknown sample could lead to gender misidentification in the absence of corroborating information. The absence of the Y allele from a known male sample could be due to either a primer binding site variant or in some cases to large deletions on the Y-cromosome [21, 25–28]. As shown in Fig. 11, the GlobalFiler™ Kit mitigates this risk by including two additional male identification markers, a Y indel and the Y-STR, DYS391, both located on the opposite arm of the Y-chromosome with respect to the Amelogenin gene.

### Color balance

**Fig. 12:**
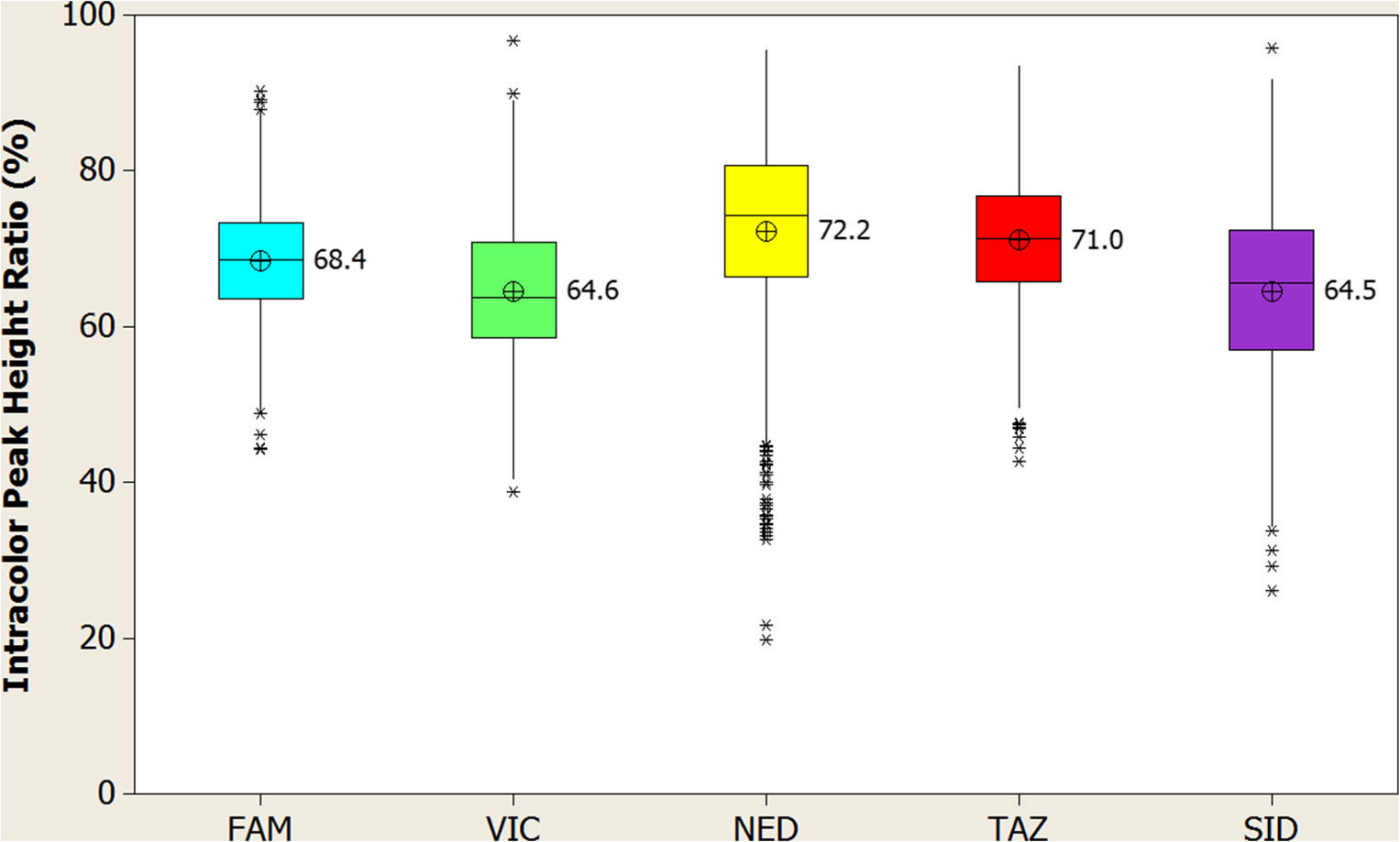
Intra-color peak height ratio analysis. Intra-color peak height ratio for each dye channel observed in population study (*N* = 817 individuals). Individual dye channels are indicated along the x-axis, with intra-color peak height ratios represented by corresponding box plots and mean indicator (circle with “x”) scaled to the y-axis. Each colored box represents the 25th through the 75th percentile of the observed values with the center line indicating the median. Whiskers indicate the range of the observed data, with points that are different than the mean by more than twice the pooled standard deviation shown as asterisks

Color balance was calculated from the later population study done to validate stutter values (see Stutter section in Results and Materials and Methods). Profiles were analyzed for heterozygote peak height ratios (at appropriate loci), and intra-color peak height ratios for each dye channel, with both values being expressed as a percentage. Peak heights within dye channels collectively were well balanced, with intra-color peak height ratio means (ICBs) over the 5 dye channels ranging from 64.5% (SID™) to 72.2 (NED™) (Fig. 12). Heterozygote peaks were also very well balanced, with mean average ratios above 80% for all alleles (data not shown).

## Conclusion

The GlobalFiler™ Kit was developed in response to demand for a multiplex assay that would capture an internationally relevant set of loci. This study evaluated the performance of the GlobalFiler™ Kit by thorough examination of the various parameters optimized by its design and protocol. In addition to attributes such as higher sample input volume and faster PCR cycling conditions than NGM SElect™ and Identifiler™ Plus kits, a number of other characteristics are noteworthy. In the GlobalFiler™ marker set, for example, the traditional sex-discriminating marker, Amelogenin, has been supplemented with both a Y-STR and a new Y_indel_, all within the VIC™ dye channel, to enhance efficiency in male/female donor identification. This new high level of redundancy is important, given that, as demonstrated here, null alleles can occur in Amelogenin and are problematic for conventional STR kits [29].

With regard to autosomal STR markers, the expanded loci set in the GlobalFiler™ Kit is essentially the result of the merging of a long-established set of predominantly European markers with the CODIS-based loci. Also included is the SE33 locus, which has long been used in and around central Europe, but has been less exploited elsewhere. While SE33’s size and complexity can present some challenges, they are also key attributes, in that SE33 has been shown to possess significantly more power of discrimination than any other STR studied for application in human identification [9–12].

Existing primer sequences from legacy kits were used whenever possible in the GlobalFiler™ Kit, to minimize the possibility of encountering new rare priming sequence variants present in populations of interest [16, 17]. Ancillary “degenerate” primer sequences have also been added for a number loci to capture variants identified after the initial introduction of the relevant earlier Applied Biosystems kits that share primer sequences present in The GlobalFiler™ Kit [13]. This current work presents evidence of good concordance for The GlobalFiler™ kit.

In conclusion, the data presented in this study clearly demonstrate the reliability, efficacy and suitability of the GlobalFiler™ Kit for forensic casework sample analysis. While this report documents the capabilities of this assay in a substantial set of experimental studies, it is recommended that each end-user lab conduct internal validation studies in accordance with SWGDAM guidelines [15].

## Electronic supplementary material

Online Resource 1 (DOCX 30 kb)

Online Resource 2 (DOCX 1371 kb)

Online Resource 3 (DOCX 75 kb)

Online Resource 4 (DOCX 91 kb)

Online Resource 5 (DOCX 2313 kb)

Online Resource 6 (DOCX 2860 kb)
