# Supplementary material for: Developmental validation of GlobalFiler™ PCR amplification kit: a 6-dye multiplex assay designed for amplification of casework samples
Source: Int J Legal Med. 2018 Mar 9;132(6):1555–73. doi: 10.1007/s00414-018-1817-5 (PMC6208722; doi:10.1007/s00414-018-1817-5)
Supplement: Supplementary file 1 — (DOCX 30 kb) [file 414_2018_1817_MOESM1_ESM.docx]

Online Resource 1

| Locus | Raji DNA Genotype | 007 DNA Genotype |
| --- | --- | --- |
| D3S1358 | 15, 16 | 15, 16 |
| vWA | 16, 19 | 14, 16 |
| D16S539 | 8, 11 | 9, 10 |
| CSF1PO | 10, 12 | 11, 12 |
| TPOX | 8, 13 | 8 |
| Yindel | 2 | 2 |
| AMEL | X, Y | X, Y |
| D8S1179 | 14, 15 | 12, 13 |
| D21S11 | 28, 31 | 28, 31 |
| D18S51 | 17 | 12, 15 |
| DYS391 | 10 | 11 |
| D2S441 | 11, 12 | 14, 15 |
| D19S433 | 14, 14.2 | 14, 15 |
| THO1 | 6, 7 | 7, 9.3 |
| FGA | 19, 27 | 24, 26 |
| D22S1045 | 15, 18 | 11, 16 |
| D5S818 | 10, 13 | 11 |
| D13S317 | 13 | 11 |
| D7S820 | 10 | 7, 12 |
| SE33 | 19, 20 | 17, 25.2 |
| D10S1248 | 12, 13 | 12, 15 |
| D1S1656 | 14 | 13, 16 |
| D12S391 | 18, 19 | 18, 19 |
| D2S1338 | 22 | 20, 23 |

Online Resourse 1: Mixture study major contributor, minor contributor, and non-overlapping genotypes. Genotypes for two different control DNA sources (Raji DNA and 007 DNA), used for mixture studies. Control 007 DNA Genotype was the minor contributor while Raji DNA Genotype was the major contributor. Non-overlapping alleles in 007 DNA are highlighted in red and were used to assess completeness of minor donor profile recovered.

Publication:

Developmental Validation of GlobalFiler^®^ PCR Amplification Kit: A 6-dye multiplex assay designed for amplification of casework samples.

International Journal of Legal Medicine

Matthew J. Ludeman*, Chang Zhong, Julio J. Mulero, Robert E. Lagacé, Lori K. Hennessy, Marc L. Short, and Dennis Y. Wang

Thermo Fisher Scientific Inc., 180 Oyster Point Blvd., South San Francisco, CA 94080, USA

* Corresponding author. Tel: +1 650 872 7271. E-mail address: [matthew.ludeman@thermofisher.com](mailto:matthew.ludeman@thermofisher.com)
