# Supplementary material for: Developmental validation of GlobalFiler™ PCR amplification kit: a 6-dye multiplex assay designed for amplification of casework samples
Source: Int J Legal Med. 2018 Mar 9;132(6):1555–73. doi: 10.1007/s00414-018-1817-5 (PMC6208722; doi:10.1007/s00414-018-1817-5)
Supplement: Supplementary file 2 — (DOCX 1371 kb) [file 414_2018_1817_MOESM2_ESM.docx]

Online Resource 2


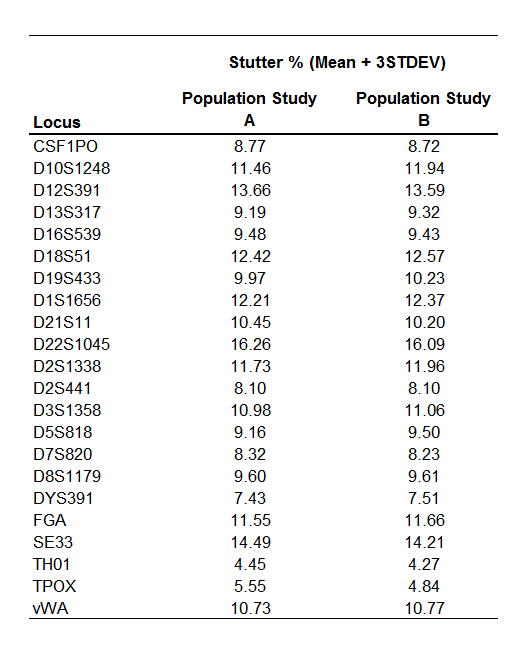


Online Resource 2: Stutter (peak at minus one repeat unit, height as a percentage of adjacent true allele peak height) levels observed, presented as Mean + 3 standard deviations for all observed stutter events (greater than 20 RFU). Population Study A was original study (N=1,094) conducted during developmental validation with early version of master mix (values are those seen in Table 4 and given in User Guide); Population Study B was an additional verification study (N=835) conducted with current on-market GlobalFiler Kit on slightly smaller subset of same donor pool. No change in values equal to or greater than 1(%) were observed, and with one exception (TPOX, -0.71), values for all markers were within 0.5(%) between studies.

Publication:

Developmental Validation of GlobalFiler^®^ PCR Amplification Kit: A 6-dye multiplex assay designed for amplification of casework samples.

International Journal of Legal Medicine

Matthew J. Ludeman*, Chang Zhong, Julio J. Mulero, Robert E. Lagacé, Lori K. Hennessy, Marc L. Short, and Dennis Y. Wang

Thermo Fisher Scientific Inc., 180 Oyster Point Blvd., South San Francisco, CA 94080, USA

* Corresponding author. Tel: +1 650 872 7271. E-mail address: [matthew.ludeman@thermofisher.com](mailto:matthew.ludeman@thermofisher.com)
