# Supplementary material for: Developmental validation of GlobalFiler™ PCR amplification kit: a 6-dye multiplex assay designed for amplification of casework samples
Source: Int J Legal Med. 2018 Mar 9;132(6):1555–73. doi: 10.1007/s00414-018-1817-5 (PMC6208722; doi:10.1007/s00414-018-1817-5)
Supplement: Supplementary file 3 — (DOCX 75 kb) [file 414_2018_1817_MOESM3_ESM.docx]

Online Resource 3.


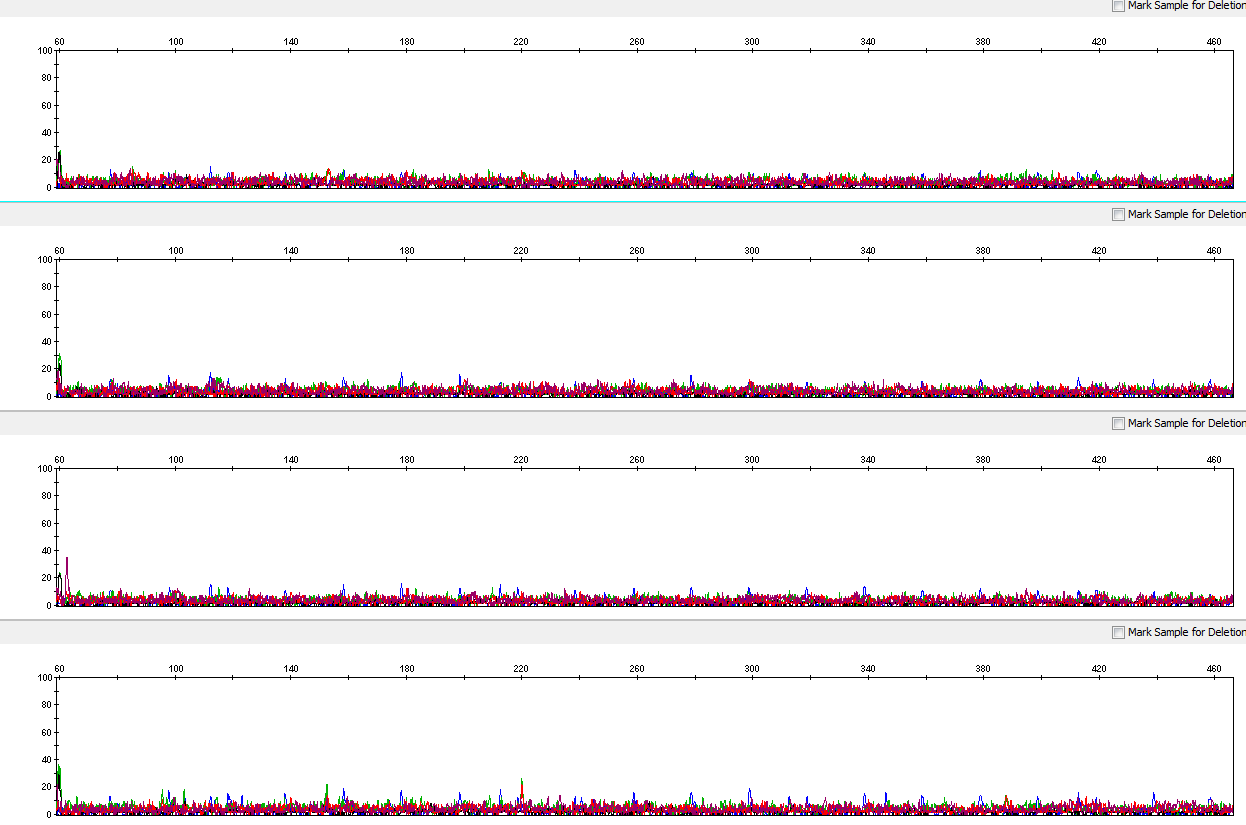


Online Resource 3. Non-temple control runs. The Y-axis spans 0 to 100 RFU. Shown are four non-template control (NTC) runs with all dye channels overlaid in each panel. Very low baseline is seen in the GlobalFiler™ Kit read region of 74 to 444 nt.

Publication:

Developmental Validation of GlobalFiler^®^ PCR Amplification Kit: A 6-dye multiplex assay designed for amplification of casework samples.

International Journal of Legal Medicine

Matthew J. Ludeman*, Chang Zhong, Julio J. Mulero, Robert E. Lagacé, Lori K. Hennessy, Marc L. Short, and Dennis Y. Wang

Thermo Fisher Scientific Inc., 180 Oyster Point Blvd., South San Francisco, CA 94080, USA

* Corresponding author. Tel: +1 650 872 7271. E-mail address: [matthew.ludeman@thermofisher.com](mailto:matthew.ludeman@thermofisher.com)
