# Supplementary material for: Developmental validation of GlobalFiler™ PCR amplification kit: a 6-dye multiplex assay designed for amplification of casework samples
Source: Int J Legal Med. 2018 Mar 9;132(6):1555–73. doi: 10.1007/s00414-018-1817-5 (PMC6208722; doi:10.1007/s00414-018-1817-5)
Supplement: Supplementary file 4 — (DOCX 91 kb) [file 414_2018_1817_MOESM4_ESM.docx]

Online Resource 4


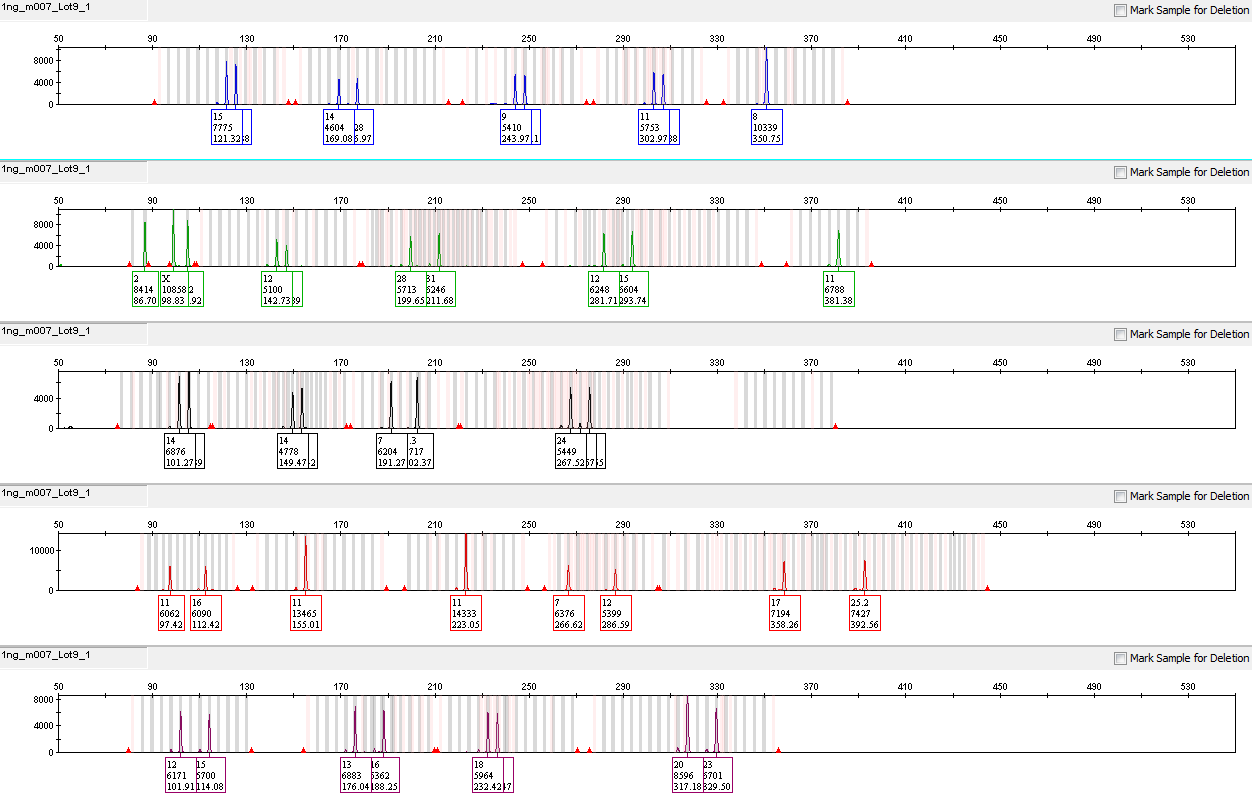


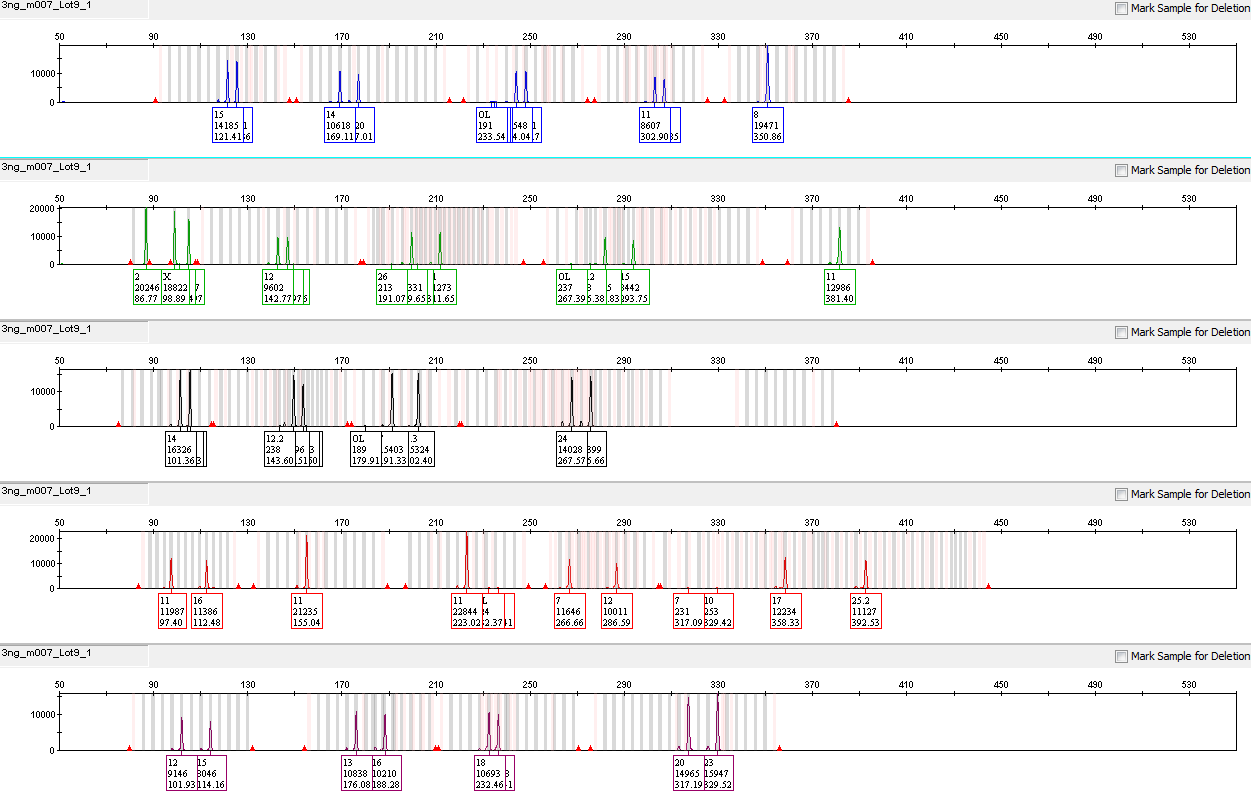


Online Resource 4. Electropherograms of amplified Control DNA 007 at 1 ng (upper panel) and 3 ng input (lower panel).

Publication:

Developmental Validation of GlobalFiler^®^ PCR Amplification Kit: A 6-dye multiplex assay designed for amplification of casework samples.

International Journal of Legal Medicine

Matthew J. Ludeman*, Chang Zhong, Julio J. Mulero, Robert E. Lagacé, Lori K. Hennessy, Marc L. Short, and Dennis Y. Wang

Thermo Fisher Scientific Inc., 180 Oyster Point Blvd., South San Francisco, CA 94080, USA

* Corresponding author. Tel: +1 650 872 7271. E-mail address: [matthew.ludeman@thermofisher.com](mailto:matthew.ludeman@thermofisher.com)
