# Supplementary material for: Developmental validation of GlobalFiler™ PCR amplification kit: a 6-dye multiplex assay designed for amplification of casework samples
Source: Int J Legal Med. 2018 Mar 9;132(6):1555–73. doi: 10.1007/s00414-018-1817-5 (PMC6208722; doi:10.1007/s00414-018-1817-5)
Supplement: Supplementary file 5 — (DOCX 2313 kb) [file 414_2018_1817_MOESM5_ESM.docx]

Online Resource 5


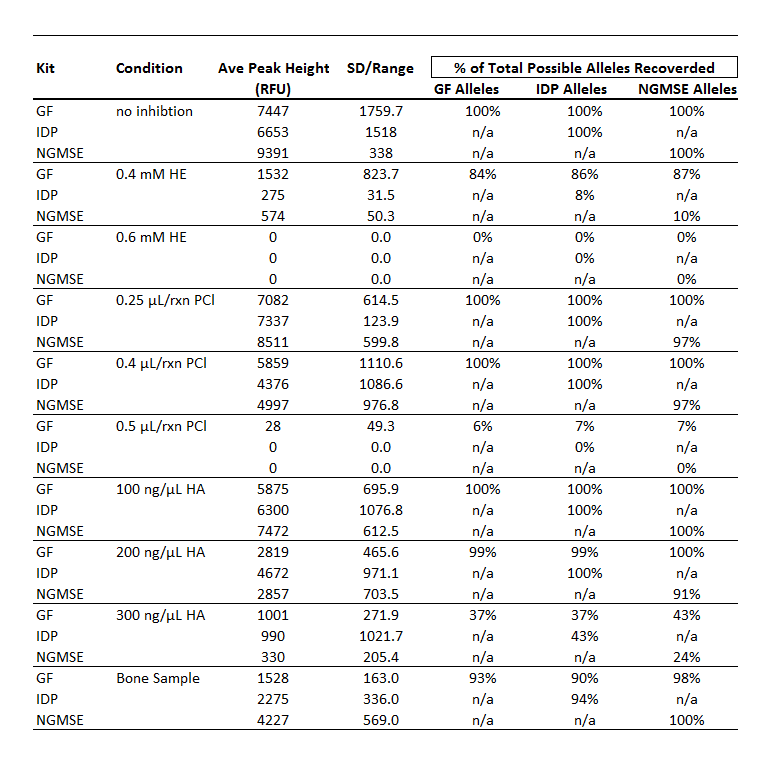


Online Resource 5. Alleles called with inhibited samples and bone samples. Percentage of alleles captured by GlobalFiler™ kit (“GF”) out of all possible alleles present in that kit as well as the two legacy kits, Identifiler™ Plus kit (“IDP”) and NGM SElect™ kit (“NGMSE”) are presented. Data on average peak heights per sample are also presented. For non-bone samples: Average Peak Heights presented in the table are the mean of three average profile peak height values from three replicates run for each kit under each condition (with standard deviation in adjacent column). Inhibitor concentrations presented are final reaction concentrations, and labels are defined as follows: HE = Hematin; PCl = Phenol-Chloroform; HA = Humic Acid. For bone sample: Average Peak Heights presented in the table are the mean of two average profile peak height values from two replicates runs for each kit, with variability presented as the range (1/2 the difference between the two average profile peak height values).

Publication:

Developmental Validation of GlobalFiler^®^ PCR Amplification Kit: A 6-dye multiplex assay designed for amplification of casework samples.

International Journal of Legal Medicine

Matthew J. Ludeman*^1^, Chang Zhong^1^, Julio J. Mulero^1^, Robert E. Lagacé^1^, Lori K. Hennessy^1^, Marc L. Short^1^, and Dennis Y. Wang^2^

**^1^**Thermo Fisher Scientific Inc., 180 Oyster Point Blvd., South San Francisco, CA 94080, USA

**^2^**Spring Bioscience, 4300 Hacienda Dr,, Pleasanton, CA 94588, USA

* Corresponding author. Tel: +1 650 872 7271. E-mail address: [matthew.ludeman@thermofisher.com](mailto:matthew.ludeman@thermofisher.com)
