# Supplementary material for: Developmental validation of GlobalFiler™ PCR amplification kit: a 6-dye multiplex assay designed for amplification of casework samples
Source: Int J Legal Med. 2018 Mar 9;132(6):1555–73. doi: 10.1007/s00414-018-1817-5 (PMC6208722; doi:10.1007/s00414-018-1817-5)
Supplement: Supplementary file 6 — (DOCX 2860 kb) [file 414_2018_1817_MOESM6_ESM.docx]

Online Resource 6


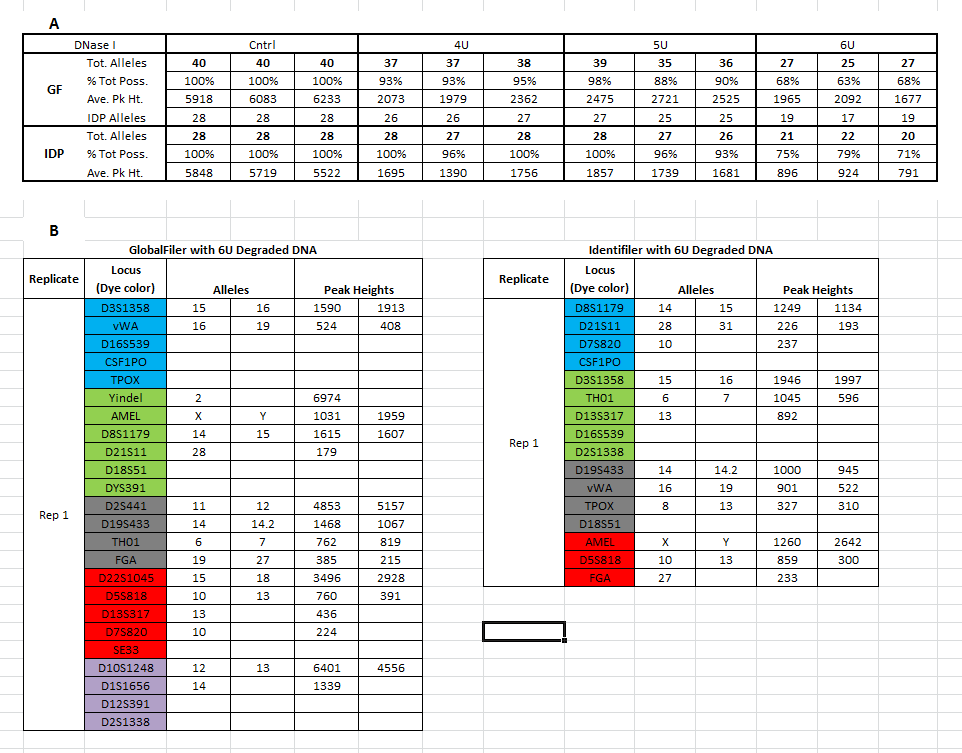


Online Resource 6.

1. Alleles capture and average peak height per profile seen with GlobalFiler™ kit (“GF”) and Identifiler™ Plus kit (“IDP”) kits with degraded input DNA. Percentage of alleles captured out of all possible alleles for each kit as well as the number of legacy kit (Identifiler™ Plus) alleles capture by GlobalFiler™ kit (“IDP Alleles”) are presented. Input DNA was pre-digested with 0 (“Control”), 4, 5 and 6 Units of DNase 1.
2. Specific loci recovery with highly degraded input DNA. Results showing specific alleles and allele peak heights called in one replicate run for each kit with the most highly degraded DNA (6 Units), representative of three replicates runs total for each kit, are presented (blank indicates no allele recovered).

Publication:

Developmental Validation of GlobalFiler^®^ PCR Amplification Kit: A 6-dye multiplex assay designed for amplification of casework samples.

International Journal of Legal Medicine

Matthew J. Ludeman*^1^, Chang Zhong^1^, Julio J. Mulero^1^, Robert E. Lagacé^1^, Lori K. Hennessy^1^, Marc L. Short^1^, and Dennis Y. Wang^2^

**^1^**Thermo Fisher Scientific Inc., 180 Oyster Point Blvd., South San Francisco, CA 94080, USA

**^2^**Spring Bioscience, 4300 Hacienda Dr,, Pleasanton, CA 94588, USA

* Corresponding author. Tel: +1 650 872 7271. E-mail address: [matthew.ludeman@thermofisher.com](mailto:matthew.ludeman@thermofisher.com)
